# Evolution before genes

**DOI:** 10.1186/1745-6150-7-1

**Published:** 2012-01-05

**Authors:** Vera Vasas, Chrisantha Fernando, Mauro Santos, Stuart Kauffman, Eörs Szathmáry

**Affiliations:** 1 Departament de Genètica i de Microbiologia, Grup de Biologia Evolutiva (GBE), Universitat Autònoma de Barcelona, 08193 Bellaterra, Barcelona, Spain; 2 Institute of Biology, Eötvös University, 1/c Pázmány Péter sétány, H-1117 Budapest, Hungary; 3 School of Electronic Engineering and Computer Science (EECS), Queen Mary, University of London, Mile End Road, London E1 4NS, UK; 4 Department of Informatics, University of Sussex, Falmer, Brighton, BN1 9RH, UK; 5 Institute for Biocomplexity and Informatics, University of Calgary, Alta T2N, Canada; 6 Parmenides Foundation, Kirchplatz 1, D-82049 Munich/Pullach, Germany

**Keywords:** origin of life, prebiotic evolution, chemical evolution, catalytic reaction networks, autocatalytic sets, replicators, protocells, metabolism-first theory of origin of life

## Abstract

**Background:**

Our current understanding of evolution is so tightly linked to template-dependent replication of DNA and RNA molecules that the old idea from Oparin of a self-reproducing 'garbage bag' ('coacervate') of chemicals that predated fully-fledged cell-like entities seems to be farfetched to most scientists today. However, this is exactly the kind of scheme we propose for how Darwinian evolution could have occurred prior to template replication.

**Results:**

We cannot confirm previous claims that autocatalytic sets of organic polymer molecules could undergo evolution in any interesting sense by themselves. While we and others have previously imagined inhibition would result in selectability, we found that it produced multiple attractors in an autocatalytic set that cannot be selected for. Instead, we discovered that if general conditions are satisfied, the accumulation of adaptations in chemical reaction networks can occur. These conditions are the existence of rare reactions producing viable cores (analogous to a genotype), that sustains a molecular periphery (analogous to a phenotype).

**Conclusions:**

We conclude that only when a chemical reaction network consists of many such viable cores, can it be evolvable. When many cores are enclosed in a compartment there is competition between cores within the same compartment, and when there are many compartments, there is between-compartment competition due to the phenotypic effects of cores and their periphery at the compartment level. Acquisition of cores by rare chemical events, and loss of cores at division, allows macromutation, limited heredity and selectability, thus explaining how a poor man's natural selection could have operated prior to genetic templates. This is the only demonstration to date of a mechanism by which pre-template accumulation of adaptation could occur.

**Reviewers:**

This article was reviewed by William Martin and Eugene Koonin.

## Background

There are two camps in the origin of life. The metabolism-first camp advocates consider improbable that RNA-like self-replicating polymers appeared before natural selection had operated on chemical networks [1-3], whereas genetics-first supporters find implausible the idea that molecular networks without genetic control could have undergone Darwinian evolution [4]. This Gordian knot was obviously cut on Earth around 3.5 billion years ago or even earlier [5]. A solution to the conundrum can be found in general evolutionary principles shared by some chemical and biological systems.

A unifying theme in both camps has been self-sustained autocatalysis: Nature's ability to generate copies of a given entity leading to multiplication. It boosted the concentration of required molecules in Darwin's 'warm little pond' [6] (or any relevant environment for chemical evolution), and allowed the increase of replicating (reproducing) units in a geometrical progression that makes the 'survival of the fittest' possible [7,8]. Dyson [9,10] and Kauffman [1,11] had developed some early ideas on how sets of mutually autocatalytic biopolymers could undergo replication, even if none of the components were individually autocatalytic. The central thesis in this scenario was that template replication is not required to achieve an autocatalytic set [12]. The various - and sometimes inconsistent - definitions of autocatalytic sets are reviewed in Additional file 1. This paper outlines a general approach that enables a more rigorous study of the structure and evolutionary potential of chemical reaction networks [13].

Originally autocatalytic sets of polymers [1,11] were introduced to explain how self-organization could provide a complex system upon which natural selection could act. It has been suggested that "...some autocatalytic sets will reproduce more rapidly than others and hence will have higher Darwinian fitness" and, therefore, "we have evolution without a genome"([1], pp. 332-333). However, no rigorous analysis of the putative evolvability of autocatalytic sets (but see [4] for a demonstration of the absence of Darwinian evolution in other models) has been carried out so far. For example, Bagley and Farmer [14] suggest that reaction networks provide a simple model for studying evolution with an emergent notion of fitness (i.e., they are clearly referring to Darwinian evolution), but subsequently [15] they used the word evolution in the Spencerian sense of change, rather than of the actual accumulation of adaptations, which is what we are really interested in.

At the time of writing then, we are still a long way from knowing whether autocatalytic sets offer a plausible model for the emergence of evolvability. The importance of such an investigation is emphasized by recent findings showing that conceptually similar models [16] failed to pass the ultimate test [4]. As John Maynard Smith [17] put it: a population of units of evolution is "any population of entities with the properties of multiplication (one entity can give rise to many), variation (entities are not all alike, and some kinds are more likely to survive and multiply than others), and heredity (like begets like) will evolve. A major problem for current evolutionary theory is to identify the relevant entities''. Can autocatalytic sets, as originally conceived, be units of evolution? Our answer is no. However, using the minimal model of polymer chemistry as a popular example, we outline the very general requirements that enable reaction networks to act as units of evolution, whatever the underlying chemistry used. We show that very simple and general network level constraints must be satisfied for evolvability in chemical networks, thus justifying the utility of such an abstract model.

In terms of "real" chemistry we have been inspired by the concept of autocatalytic protein networks [1,10,18], but in principle several different polymer species (even RNA) could potentially realize such networks. In fact, a similar evolutionary mechanism has even been demonstrated in a model of random bimolecular rearrangements rather than ligation and cleavage reactions, thus extending the scope of the mechanism proposed here [19,20]. One may object to the motivation of the present paper by simply pointing out that no autocatalytic polymer networks have been shown to exist yet, let alone to evolve. This criticism is not completely fair, since we have the autocatalytic networks of peptides realized by Ghadiri and Ashkenazy [21] (although there a direct templating effect as in [22] plays a crucial role, while the polymer chemistry we assume here does not rest on direct template replication). In any case, it is a fact that amino acids and protein(-like) polymers readily form in various prebiotic experiments, and random polypeptides show all kinds of spontaneous enzymatic activity, whereas we certainly do not know where the RNA world came from. What we are interested in is whether spontaneous enzymatic activity of such polymers can "ignite" reflexively autocatalytic networks, and whether these networks can undergo evolution by natural selection. This theoretical analysis is not a substitute for badly needed experimental work, not even for more detailed theoretical analyses: it is a first step in a (surprisingly) promising direction, and aims to stimulate experimental and further theoretical work.

The first part of the paper examines the original claims made about autocatalytic sets, discusses earlier criticisms of these models, and identifies various chemical organizations within the reaction network. We focus on an entity we call the autocatalytic core. The second part dismisses the putative evolvability of autocatalytic sets *per se*, but proves the capacity of certain special kinds of chemical reaction networks, i.e. those containing multiple autocatalytic cores enclosed in compartments, to sustain selectable hereditary variation. It is essential to realize that there has been no demonstration previously of how or even whether chemical reaction networks could accumulate adaptations.

## Results and discussion

### Anatomy of an autocatalytic set

Kauffman ([11] pp. 2-3) defines an autocatalytic set as an arrangement of molecules in which "every member of the autocatalytic set has at least one of the possible last steps in its formation catalyzed by some member of the set, and that connected sequences of catalyzed reactions lead from the maintained 'food set' to all members of the autocatalytic set". This is more formally defined by Hordijk and Steel [23,24], who state that an *R *(sub)set of reactions is called (*i*) reflexively autocatalytic (RA) if every reaction in *R *is catalyzed by at least one molecule involved in any of the reactions in *R*, (*ii*) food-generated (*F*) if every reactant in *R *can be constructed from a small "food set" by successive applications of reactions from *R*, and (*iii*) reflexively autocatalytic and food-generated (*RAF*) if both *RA *and *F*.

**Figure 1:**
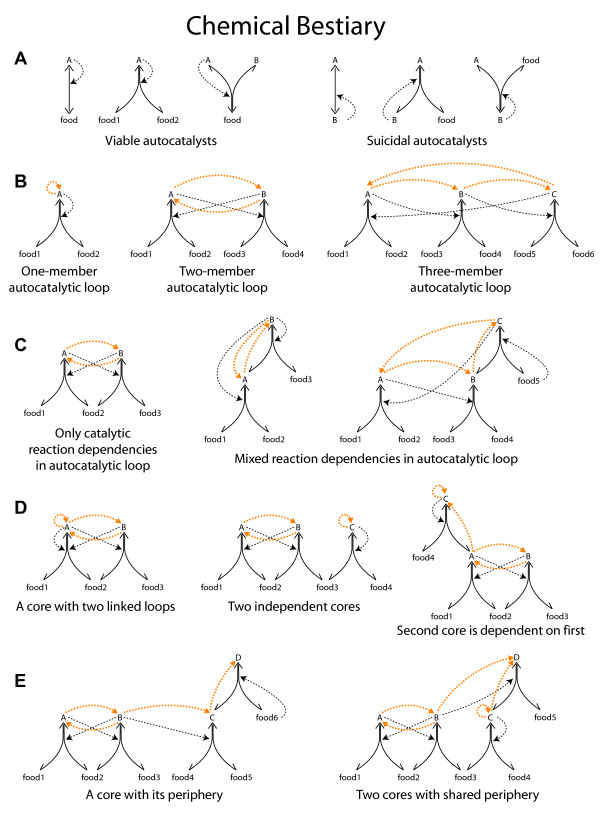
**Classification of various network modules within autocatalytic sets**. food1-food6: food set that is assumed to be present at all times, A-D: non-food species generated by ligation/cleavage reactions. Solid lines: reactions; dotted lines: catalytic activities. Orange dotted lines show the superimposed autocatalytic loops. (***A***) Viable autocatalysts (A in all three examples) are the necessary units needed for exponential growth of an autocatalyst, in contrast to suicidal autocatalysts (B in all three examples) that use reactants only produced by the autocatalytic reaction itself. (***B***) A molecular species can be directly autocatalytic, forming a one-member autocatalytic loop, or several species can form loops of various sizes that result in indirect autocatalysis. (***C***) A loop is autocatalytic - and able to grow exponentially - as long as at least one of the steps is a catalytic dependency. Therefore a loop can be made of solely catalytic or mixed couplings. (***D***) An autocatalytic core contains one or more linked loops. Note that any member of a core (A and B in all three examples) is sufficient to act as a seed for the core. Several distinct cores can form within a catalytic reaction network. Some can exist independently of other cores, while dependent cores rely on others as food supply or catalysts. (***E***) An autocatalytic core is typically associated with a periphery that is dependent on the core (C and D in first example). It is also possible that a molecular periphery appears only if two or more cores are present (D in second example). We propose that autocatalytic cores are the units of heritable adaptation in chemical networks.

**Figure 2:**
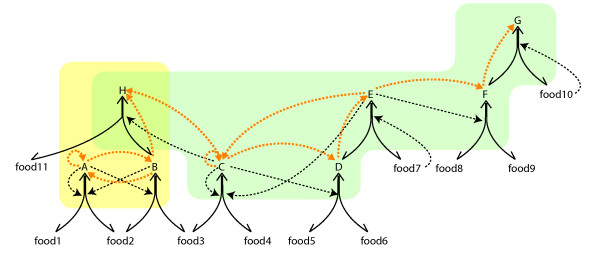
**Multiple cores result in selectable attractors for a chemical network**. food1-food11: food set that is assumed to be present at all times, A-H: non-food species generated by ligation/cleavage reactions. Solid lines: reactions; dotted lines: catalytic activities. Orange dotted lines show the superimposed autocatalytic loops. *Structural considerations: *Autocatalytic sets can contain several distinct autocatalytic units, each of which can be divided to a core of autocatalytic molecules and a periphery. Here, two independent cores are shown. The first consists of the two linked loops A → A and A → B → A. The second core includes the two linked loops C → C and C → D → E → C, with the periphery of F and G. H is the shared periphery of the two cores that requires both for its production. *Dynamical considerations: *This platonic reaction network can manifest in four possible stable compositions of the core-periphery units: (i) no cores (only food species); (ii) only first core (A, B; yellow area); (iii) only second core (C, D, E, F, G; blue area); (iv) both cores (all species). Now imagine that we have a compartment that only contains food species, but rare uncatalyzed reactions among them are possible. The uncatalyzed appearance of any one molecule of core species is sufficient to produce all the core species and the periphery species of that core, e.g. either A or B for the first core and either C, D, E for the second. Now let us assume that after reaching a certain size a compartment that contains both cores will split and produce propagules. If neither C, D or E is present in the daughter compartment, the second core is lost and the remaining molecules of its periphery will be washed out from the compartment. Discovering cores by rare reactions, and losing cores by segregation instability opens up the possibility for a chemical reaction network to respond to natural selection.

The concept of an autocatalytic or RAF set, although important for questions of self-organization, does not directly address heredity or selectability [24]. However, such a set can be divided into one or more strongly connected autocatalytic cores and their peripheries, and we propose that these cores are the units of heritable adaptations in reaction networks (for chemical network motifs see Figure 1 and for a specific example see Figure 2). A core can be viewed as a chemical network genotype and its corresponding periphery as a chemical network phenotype (although without a modular or compositional mapping between them). An autocatalytic core (which we abbreviate from now on to 'core') contains one or more linked autocatalytic loops [18]. Autocatalytic loops are closed circular paths of any length where each molecule in the loop depends on the previous one for its production (Figure 1). In the core all species catalyze the production of all other species, including themselves, which means that they are indirectly autocatalytic (Figure 1). The periphery consists of molecular species that are catalyzed by the core (Figure 1). The provision of any one molecule of core species is sufficient to produce all the core species and the periphery species of that core; in other words, all core molecules contain the information that is necessary for igniting and sustaining the autocatalytic core and periphery and can therefore act as an autocatalytic seed. This is not the case for periphery molecules that depend, as a phenotype does on its genotype, upon the core. Note that an autocatalytic or RAF set as defined above can contain any number of distinct core-periphery units (Figure 1) and the structurally and kinetically possible combinations of such units define different alternative stable states of the same chemical network (Figure 2).

Having defined an autocatalytic core as a set of connected autocatalytic loops, it is important to distinguish between the possible types of such loops (Figure 1). Typically, the cycle of reactions is coupled by catalytic dependencies. However, as Eigen [18] has shown, the cycle maintains its autocatalytic properties as long as at least one of the steps is catalytic (an idea that was missed by previous models in [11,23]). All other steps can be substrate dependencies where the product of the previous step serves as a precursor for the next reaction (e.g. consider the dependency of Y upon X in the reaction A + X ←→ Y; Figure 1). In other words, one reaction where the product is not consumed but serves as a catalyst is enough for the exponential increase of the mass of the cycle. It can be misleading to solely focus on directly or indirectly autocatalytic molecules also, because of the possibility of what we call suicidal autocatalysts [13]. An autocatalytic molecule can be suicidal in a kinetic sense. Bearing in mind that all reactions in autocatalytic sets of biopolymers [11] are assumed to be reversible, let us consider the simple autocatalytic reaction A + X ←→ 2A. If X is not present (or present in very low concentration) this reaction will go in the direction of self-decomposition, in a form of autoinhibitive cycle (Figure 1). Such suicidal autocatalysts are obviously incapable of exponential growth, the very feature that gives autocatalysis its evolutionary significance. A viable autocatalytic molecule, either directly autocatalytic or embedded in a longer autocatalytic loop, however, does grow exponentially. Note that rather similar examples of viable and suicidal autocatalysts can be found in contemporary biochemistry: whereas the Calvin cycle is a network of autocatalytic sugar production, the pentose phosphate pathway is an example of autoinhibitive decomposition of sugar phosphates [25]. Viable autocatalytic loops are necessary but not sufficient for evolution by natural selection of autocatalytic networks, as we shall see below.

### Spontaneous formation of autocatalytic sets in a polymer chemistry

The original mathematical model of autocatalytic sets [11] assumes the following: (*i*) there exists a large food set of abundant polymers naturally formed in the environment up to some low level of complexity, i.e. up to length *M *consisting of *B *types of monomers (e.g. **a, b, aa, bb**); (*ii*) each molecule has a certain probability *P *of catalyzing each ligation-cleavage reaction. The model assumes infinite discriminability, in other words, a molecule either does or does not catalyze a particular reaction without quantitative variation in efficiency. However, it does not assume specificity, because a catalyst typically catalyses a number of reactions (on average, *P *fraction of the possible reactions). It was demonstrated that above a certain catalytic probability threshold a chain reaction is triggered and due to catalytic closure autocatalytic sets appear [11].

**Figure 3:**
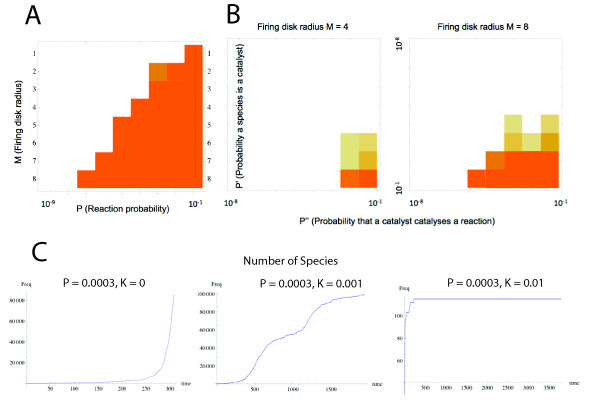
**Emergence of a self-sustaining network of reactions in a flow reactor**. (***A***) The squares show critical thresholds for subcritical (empty squares) or supracritical (coloured squares) growth of the reaction network as a function of the firing disc (maximum length of molecular species in the food set) and the probability *P *that a species catalyses a specific reaction. The darkness of a square reflects the proportion of 100 runs in which the network exceeded one of the following conditions: > 2 × 10^7 ^reactions or > 10^5 ^molecular species (note that in any finite system the reaction network cannot be explored infinitely due to mass constraints). (***B***) The crucial parameter *P *was decomposed into its two elementary probabilities: *P' *(the probability that a species can be catalytic) and *P'' *(the per reaction probability that this catalyst catalyses a reaction). When *P' *decreases *P'' *must be considerably higher for reaction networks to keep growing, but there is a threshold above which catalytic networks grow supracritically. (***C***) Weak inhibition does not prevent formation of large catalytic reaction networks. For values of *P *that do produce catalytic network growth, strong non-competitive inhibition is introduced by choosing with probability *K *that a species removes another species from the reactor *completely *if at least one molecule of the inhibiting species exists (this is clearly a worst-case assumption). Left: supracritical growth without inhibition. Middle: weak inhibition results in alternating fast and slow growth phases. Right: strong inhibition makes the network subcritical.

Hordijk and Steel [23] verified this claim by generating random networks of reversible ligation/cleavage reactions between strings up to length *n *= 20, where each molecule had the probability *P *of catalyzing each reaction. At low values of *P *they found unconnected sets utilizing separate food sets, but at higher values a percolation phenomenon produced fully connected autocatalytic sets. Farmer and co-workers [12,14] were the first to implement the original mathematical model and confirmed that a supracritical reaction network that keeps growing with accelerating speed arises above a certain catalytic probability *P*_*c*_. By constraining the growing catalytic reaction network in a flow reactor with finite mass and lower bound concentration threshold (the relevant scenario being here to study the issue of evolvability in compartmentalized systems), they implemented a chemical model where the size of the chemical network shows logistic growth above *P*_*c*_. Our initial task was to corroborate these results and to investigate the underlying structure of the catalytic reaction network (the methods are described in detail in Additional file 1). We found that as the networks grow in size they form one large autocatalytic core consisting of all molecular species above the concentration threshold. However, autocatalytic cores mostly consist of suicidal autocatalysts as only a small minority of autocatalytic species use valid reactants in the autocatalytic reactions and thus form viable loops (see Figure 1). Note that although the number of viable loops increases with system size, they are always within the same viable core and therefore they cannot be independent targets for natural selection (see Additional file 1). In conclusion, we substantiate the speculation [1] that a self-sustaining network of reactions -an autocatalytic primitive metabolism- appears in this minimal model of polymer chemistry (Figure 3).

Our next task was to verify that the previous claim remained true in the face of earlier criticisms of the model. Thus, a critical parameter in the model is the probability *P *that each molecule can catalyze each ligation-cleavage reaction, which was assumed to be constant. This assumption led to the serious objection that the model implied an unrealistically high probability (of one) that a peptide could act as a catalyst [26]: when the maximum length *M *of polymers in the set increases, the number of reactions (((*M*-2) × 2^*M*+1^); [11]) increases faster than the number of molecules (*N* ≈ 2^*M*+1^), therefore all molecules quickly become catalytic - an outcome that is clearly very unrealistic. To remedy the situation, the parameter *P *should be a composite of two probabilities: the probability *P' *that the molecule is a catalyst, and the probability *P" *that a molecule catalyzes a given reaction [26]. We implemented this criticism, but with the caveat that it is unlikely that a catalyst is expected to catalyze only one out of the infinite possible reactions as suggested by Lifson [26], following assumptions in ([1] p. 306). That is, defining

(1) $$P=P^{\prime }\cdot P^{\prime \prime }=P^{\prime }/)\left[\left(M-2\right)\times 2^{M+1}\right]$$

it is implicitly asserted that the probability that a catalyst catalyzes a given reaction is not independent of the probability that it catalyzes another reaction - but why would considering a bigger reaction space make catalysis less likely? In our view, a more reasonable scenario is to assume that *P' *is defined as it was above, but *P" *is now considered to be the *per reaction *probability that a catalyst catalyzes the reaction. When our previous simulations were re-implemented with constant *P' *and *P" *values it was found that when the ratio of catalysts (*P'*) decreases, the probability of catalysis (*P"*) must be considerably higher for reaction networks to keep growing (Figure 3A and 3B). Therefore, even though there is no known random polymer chemistry in which these probabilities are ever so high as necessary for supracritical growth -certainly not random polypeptides [27]-, we conclude that Lifson's [26] criticism remains a quantitative one, leaving open the possibility that were it possible to obtain such catalysts, the catalytic network could still form spontaneously.

A second objection to the model was that autocatalytic sets could not have been formed spontaneously due to the 'paradox of specificity'; that is, a high number of molecules is required for spontaneous emergence of a self-sustaining network of reactions [1], but the harmful effect of side reactions that ought to rise with an increasing set calls for a small system size [28]. One way to check whether harmful effects of side reactions in spontaneously emerging autocatalytic sets could inhibit network growth is to introduce strong non-competitive inhibition. It is easy to imagine that a species removes another species from the reactor by some side reaction, and we chose to implement the strictest possible scenario where one molecule of inhibitor removes the inhibited species completely. In a manner analogous to the determination of catalytic reactions, each molecule inhibited any of the other molecules with the probability *K*. A species may therefore be both an inhibitor and also have other positive catalytic effects. It should be noticed that competitive inhibition already emerges in the model in the case where a catalyst uses another catalyst as a substrate, and so it is not necessary to explicitly add this. At high levels of inhibition (e.g. *K *= 0.01) the consequence is to convert what would have been a supracritical network to a subcritical one. However, at lower levels (e.g. *K *= 0.001) the effects of poisoning do not radically prevent supracritical growth, but the networks grow non-monotonically due to the loss of some catalysts because of inhibition (Figure 3C). We therefore conclude that inhibition does not qualitatively prevent formation of large catalytic reaction networks. To summarize, the formation of autocatalytic sets is robust against the two main criticisms that have been raised against the model.

**Figure 4:**
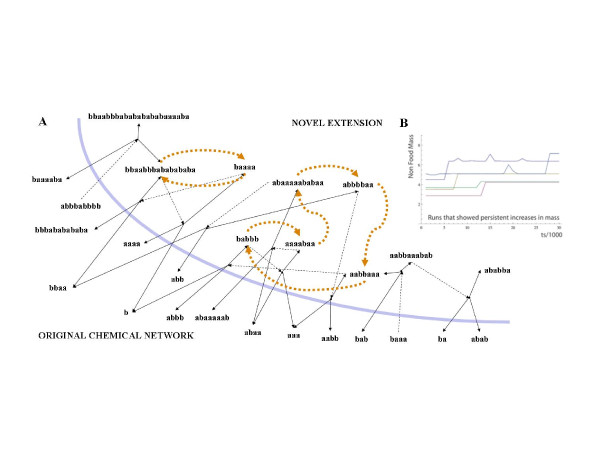
**Persistent increase in non-food set mass due to novel viable loops**. We simulated 460 runs lasting 30,000 growth steps each, with food set size *M = 4*, *P' = 0.75, P'' = 0.0025, K = 0 *(without inhibition), but with spontaneous emergence of rare novel species from uncatalyzed reactions. 5 out of 460 runs showed persistent increases in non-food set mass (***B***). This was always due to the incorporation of at least one viable loop. (***A***) Example of viable loop organization used in evolutionary simulations. Solid lines: reactions; dotted lines: catalytic activities. Orange dotted lines show the superimposed autocatalytic loop. The original network, on the left side of the blue line, is not shown in detail.

All the previous simulations assumed that only catalyzed reactions happen in the flow reactor. Bagley and co-authors [14,15] modelled the background of uncatalyzed reactions as spontaneous fluctuations that resulted in the rare appearance of autocatalytic subgraphs from the shadow of existing reactions (subset of species that can be produced from existing species in uncatalyzed reactions), an approach we find problematic because (i) it already assumes without proof that autocatalytic loops are present and (ii) it only allows for loops where each step is catalytic, discarding a large variety of possible organizations. In order to avoid this flaw, we simulated the uncatalyzed reactions directly. In our model rare uncatalyzed reactions produce random novel species in low copy number from the shadow, and if the new molecule happens to be a catalyst, it will generate a chemical avalanche of directly and indirectly catalyzed further novel molecular species. As expected, we found that only those species are able to permanently join the network that eventually catalyze their own production from already existing molecules and so produce a viable autocatalytic loop (see Figure 2). Such viable loops define a new, distinct core within the autocatalytic set. Such a novel core is only rarely produced, at least in the small networks we simulated, but the probability of their spontaneous appearance depends on the size of the shadow and is expected to increase with network size and *P*. There is, therefore, an intrinsic slow tendency for non-food set mass to increase by rare incorporation of viable loops in the network that also results in the increase of complexity (Figure 4, Additional file 1). This appearance of novel cores is a critical property as we shall see next.

### Evolvability of chemical networks enclosed in compartments

Our next step was to tackle the issue of evolvability when chemical reaction networks are confined into a small volume (compartment). Now the question is: what is required for chemical networks to undergo Darwinian evolution? As emphasized by Gánti [29] and Wächtershäuser [30,31], if *distinct*, *organizationally different*, *alternative autocatalytic *networks can coexist in the same environment then they could compete with each other and the 'fittest' would eventually prevail. This is obviously a narrow view of what a unit of evolution really is [17], but raises the important issue that reaction networks must somehow posses multiple attractors and transitions among attractors must be possible. As Wesson [32] put it, "the attractor is the essence of self-organization. Just what constitutes it and how the organism shifts from one attractor to another is a task for genetics... to elucidate". This message is even clearer in Conway Morris [33], who posits that evolution navigates to particular functional solutions (convergence) thus pointing to the existence of something analogous to 'attractors' in biological systems. We demonstrate that for a catalytic network to accumulate adaptations it needs to be compartmentalized, the platonic reaction network must have multiple attractors, and some of these attractors must be selectable. The larger the number of attractors, the smaller the chance of convergence.

Compartmentalizing the reaction networks enables filtering out harmful modifications and therefore it is a prerequisite for accumulating potential beneficial 'adaptations', as demonstrated in [19]. We modelled compartments exactly as in Farmer et al. [12]; that is, each compartment is a flow reactor in which food is input, and materials leak out at first order. The number of attractors is itself of interest, for they allow a protocell to have multiple pathways of autocatalysis and also to show molecular variability to respond to an environment. We approximated the number of attractors by fixing the reaction network and shuffling the chemical concentrations (by choosing random pairs of species and swapping their concentrations) several times in order to sample various initial conditions. After shuffling, the network dynamics are run for some fixed period of time until an attractor is reached. Even if multiple attractors exist, stochastic division might not generate sufficient variation to allow transition between them. To test this, we also simulated the more realistic situation where the compartment enclosing the generative chemistry was allowed to grow for a fixed period of time, after which it was assumed to split into daughter compartments, whose molecules were sampled from a polyhypergeometric distribution of molecular contents in the parental compartment.

Now we arrive at the critical issue of evolvability, which can be first rephrased as the potential of a population of compartmentalized molecular networks with different attractors to respond to selection; that is, to transit between different attractors according to the fitness value assigned to each of them. As a preliminary test of evolvability, reaction networks were subjected to artificial selection. A small population of 10 compartments was isolated for a fixed generation period. After this time the fitness of each compartment, defined as the total mass of non-food species present at the end of the growth phase just prior to division, was assessed and production of the next generation occurred by taking molecule propagules from compartments on the basis of fitness proportionate selection (roulette-wheel selection with elitism [34]). This elitist selection was used to always sample at least one propagule from the individual with the highest rank in any given generation.

The results of the artificial selection experiment were confirmed in numerical simulations of natural selection. Here we assumed an initial population of N = 100 compartments and introduced a classical Moran process [35] to test the evolvability of reaction networks when subjected to natural selection. Thus, in each time step a randomly chosen compartment from the whole population is selected for growing at a rate which is a function of its chemical composition. The compartment is returned to the population whenever its size is less than *η *molecules and the step ends. If, however, size reaches *η*, the compartment generates two daughter compartments by creation of two propagules. One offspring replaces the parent compartment and the other a randomly chosen one from the population. In this stochastic process, the total number of compartments remains constant and given by N, but compartment's size can fluctuate between the propagule size and *η *molecules. Selection for a specific target was implemented by multiplying the rates of all reactions by a selective advantage *S *if it matched the characteristic composition of the desired attractor.

We tested the evolvability in all three previously described models - the original Farmer-type autocatalytic sets, networks with inhibition, and networks with random novel species produced by uncatalyzed reactions - according to the principles described above. In the case of the original networks [12] the results were straightforward: they always have only one attractor (Additional file 1) and selection is not possible. This was not surprising considering that these networks contain only one autocatalytic core (Additional file 1). Therefore, the conclusion immediately follows from our previous considerations: Kauffman's [11] original polymer chemistry when enclosed in a finite space will eventually crystallize into the same attracting network which can never ever be a Darwinian unit.

Interestingly, this behaviour is analogous to conceptually similar models' [4] where the whole catalytic network forms inevitably only one viable core and so ultimately converges to only one attractor. Therefore, one important conclusion to be derived from our work is that we can definitively discard all autocatalytic networks discussed so far in the literature as units of evolution in the Darwinian sense, with the possible exception of [19].

However, it has been suggested [1] that the inclusion of inhibition in the Farmer-type network should permit the formation of autocatalytic sets having complex dynamical attractors. To determine if this is so we also run simulations introducing strong non-competitive inhibition as indicated above. Interestingly, our results substantiate this speculation because molecular networks now exhibited multiple attractors, but when the growth-splitting process was implemented spontaneous transitions between them were rare. When transitions did occur, they happened either periodically or chaotically (Additional file 1). Rather surprisingly, the artificial selection experiment excluded networks with inhibition from candidates of units of evolution, since the population typically settled down into one equilibrium or fluctuated stochastically or periodically between attractors and so attractors typically could not be stably selected (Additional file 1). Instead, the internal dynamics of the growth-splitting process completely overrode any effect of selection. This provides *a clear counter-example to the widely accepted claim that the existence of multiple attractors is sufficient to allow selectability; it is not*.

The crucial modification to the model was to allow rare novel species to appear from the shadow. In the few cases of networks in which spontaneous addition of new species resulted in the ignition of a novel viable loop, and thus novel cores, there always existed multiple attractors (see Figure 2. for a didactic example and Figure 4 for the network used in the evolutionary simulations). Note that we did not simulate inhibitory reactions in this version of the model; while they are certainly relevant in applications closer to real chemistry, their inclusion would have made our results on viable loops more difficult to interpret. Analogous to the idea that 'attractors' in biological systems have different stabilities (i.e., convergence can be equated with the revisiting of most stable attractors [33]), we also detected stable attractors (with a larger attractor basin) where the system settles most of the time with occasional transitions to less stable attractors (with smaller attractor basins).

**Figure 5:**
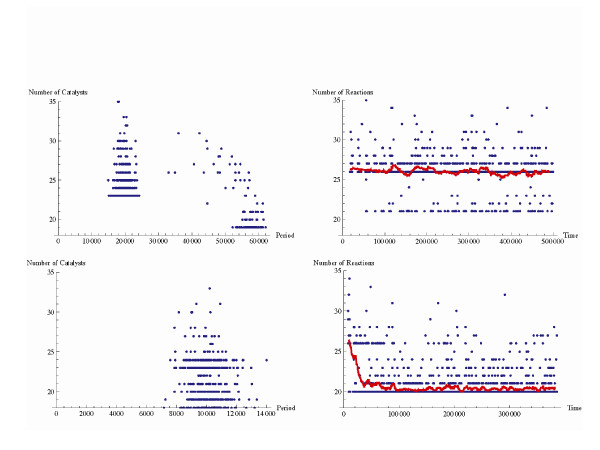
**Selectability of potentially coexisting attractors in a molecular network**. Each dot corresponds to a compartment just prior to division. (***Top***) Due to its autocatalytic properties a viable loop enables a higher growth rate and therefore the network with the large viable loop (characterized by 26 reactions and dividing after approximately 20 000 time steps) constitutes the most frequent network type. Spontaneous reaction rate = 0.00001. Propagule size 800; no selection (*S *= 1). (***Bottom***) However, with a mere 1 percent fitness advantage (*S *= 1.01) attributed to the networks without the loop, it is possible to reduce its frequency. In this case the original network without any viable loops is the most frequent.

We intuitively suspected that selection would work in networks with novel viable loops, and this was indeed the case. Our results can be summarized as follows: while networks with the viable core have an implicit selective advantage due to their higher growth rate, and so constitute the majority of the population, a one percent selective advantage attributed to the absence of the core is enough to significantly reduce the proportion of networks with viable cores in the population (Figure 5). The reason for the selectability in this model is that a novel viable core results in a new and distinct attractor for the reaction network, and due to its autocatalytic properties enables a higher (non-food mass) growth rate. Hence, we already have the basic requirements for natural selection to happen: two entities that are growing exponentially at different rates and have different division times [36]. Since it is always possible to lose the viable core upon protocell fission (a loss mutation that is simply a function of propagule size), there is a kind of 'mutation-selection' balance if no novel chemical species can invade from the shadow. When rare reactions are allowed, novelty can arise by generation of new viable cores, and they can be removed by selection if they reduce the growth rate of the compartment. Between-compartment selection as shown in Figure 5 arises due to the effect a core has on the compartment level fitness. For example, the large core (Figure 4) sustains more non-food mass in its core and its periphery, and this increases the growth rate of the compartment. In reality, each molecule of the core and its periphery may confer a host of compartment level effects, e.g. modification of permeability of the membrane, specific metabolic adaptations, etc. that could have a compartment level fitness effect, but this is not explicitly modelled here.

It is important to note that there are two levels of autocatalysis in this system. Even if the internal organization of the network encapsulated by a protocell fails to be autocatalytic, the rule that after reaching the critical mass the compartment divides into two effectively ensures that such compartments will have a 'generation time' and the potential to grow exponentially. Also autocatalytic cores grow exponentially. Hence there is autocatalysis at two levels: the level of molecules and that of compartments. The reproducing compartment without an enclosed autocatalytic network is not, however, a replicator, as it always assumes the same state and cannot sustain hereditary variation [28].

## Conclusions

It should come as no surprise, our finding that independent viable autocatalytic cores embedded in a large molecular network can be considered as units of evolution, since the basic ingredients of differential growth rates and division times among potentially competing entities are fulfilled. The reader might, however, query that we have not properly addressed the issue of evolvability because no mention of heredity has been made. To fully understand that this criticism does not apply here it is important to appreciate the implications of the core-periphery dichotomy in autocatalytic sets. This dichotomy can be translated into a kind of genotype-phenotype mapping in fully fledged biological systems and, interestingly, allows us to appropriately use the terms replication and reproduction despite the fact that we are dealing with an assembly of molecules [37]. Thus, the viable cores could be considered the units that replicate and, once transmitted to the offspring compartments after the parental compartment splits (reproduces), they give raise to the same periphery; that is; there is a clear matching between a viable core ('genotype') and the periphery ('phenotype'). 'Mutation' happens either when uncatalyzed reactions result in the emergence of a novel core, or when molecular components of a viable core are stochastically lost after compartment splitting. Thus, our autocatalytic networks are capable of stably transmitting information across generations [17].

However, a viable core constitutes one bit of heritable information and therefore the number of possible selectable attractors is relatively small, meaning that autocatalytic networks may not be able to sustain open-ended evolution. While we think this to be the case, the potential role of these autocatalytic networks as a route to nucleotide-based template self-replicating systems should not be underestimated. The chemical reaction networks show an intrinsic tendency to increase in complexity. Whenever novel spontaneous reactions occur, the number of possible uncatalyzed reactions also increases, opening up new possibilities for discovering viable cores (genotypes) and their corresponding peripheries (phenotypes). This 'cooptive evolution' [13] involves stepwise expansions into (and retractions from) the adjacent possible of reaction space [38].

It is important to note that we do not claim that the present work renders the RNA world obsolete. In fact several of the authors of the present paper have worked under the assumption that indeed there had once been an RNA world. But this does not mean two things: first, that the RNA world was "clean" (probably it was not; other molecules, large and small, are likely to have been around and to have served even key functions), second, that reflexively autocatalytic networks could not have preceded the RNA world (they may have been indispensable "scaffolds", *sensu *Cairns-Smith, [39] for its appearance). As suggested by Dyson [10], RNA could have entered as a kind of waste/parasite, but already under the catalytic influence of *evolved *(as we argue) autocatalytic networks. Genetic takeover does not in principle require reverse translation or any similar esoteric process; one just needs room for stepwise innovation and improvement.

Our results should be contrasted with the lipid world scenario [4,16,40] that so far has failed to offer models that would demonstrate a capacity for evolvability. The problem is that the simplicity of the underlying chemistry in GARD (lipid molecules are either in the assembly or not) allows only as many reactions as there are different molecular species available in the environment. Moreover, the number of distinct lipid types cannot be too high, partly because of practical considerations, but also because increasing diversity implies increasing noise in compotype replication. The restricted diversity of molecules and reactions means that the system will always quickly converge to the state set in stone by the underlying dynamical equations [4]. The only possibility left open for change is the addition or removal of lipid species. Pointing out that altering the food set of a reaction network modifies its dynamics, however, has no relevance for evolution. A combinatorial chemistry like the polymer chemistry described in this article, on the other hand, provides an unlimited diversity of theoretically possible reactions originating from the same food set and a reasonable probability that a reaction network can discover novel cores in its shadow. Also, the permanent incorporation of a new core will extend the shadow, opening up new possibilities. Therefore we argue that such a combinatorial chemistry (or one with similar complexity) is essential for even limited evolution. The complexity of a lipid world is overshadowed by the possibilities enabled by the outlined polymer chemistry, which itself is only a shadow of the world of template-replicating nucleic acids.

We stress that there is a crucial difference between small-molecule autocatalytic cycles (such as the reductive citric acid cycle) and reflexively autocatalytic sets of polymers. First, a family of polymers (such as proteins) can be synthesized by a small set of canonical chemical reactions, whereas the reductive citric acid cycle consists of chemical steps of various kinds (cf. Orgel [41]), thus the former can more readily be catalyzed by environmental (i.e. unevolved) catalysts. Second, and more important, polymers can, due to their modular construction, show targeted and specific activity in catalytic task space. The increased efficiency of catalysis carries over to resistance against side reactions [41] that constantly divert material from useful pathways. However, these facts merely change the probability of formation of viable cores in particular chemical systems, not the fact that Darwinian evolution is possible once they appear.

The remaining open issues are experimental and theoretical in nature. We need better models and, above all, relevant experiments. The systematic consideration of the experimental realization and evolvability of autocatalytic networks of small organics (such as those of intermediate metabolism [2,19,29,31]) require further scrutiny in the light of the proposed selectability principles. It is not farfetched to claim that an empirical scientific program implementing the sort of simple chemistry used in these models is worth pursuing. The recent calculations of Amend and McCollom [42] indicate that amino acid production in ancient hydrothermal vents could have been thermodynamically favoured, providing a continuous supply of monomers for the hypothesized peptide network. Autocatalytic networks of peptides already have been synthesized by Ghadiri and Ashkenazy [21], although there a direct templating effect plays a crucial role. Protein networks that do not employ templating are more difficult to realize, but several recent advances hint they might be possible. There exists a dipeptide that does catalyze ligation of peptides [43]. Random peptides of length about 32 and 74, biased to known ratios of amino acids in evolved proteins, fold into compact structures for 30% of such sequences [44], and longer random polypeptides have shown to have catalytic activity [45]. The folding and catalytic properties of "never before born" peptides is therefore an open experimental question that could be addressed with random peptide libraries - it is a project much to be sought, and we should make it clear that these experiments are now needed, and hopeful given the promising results cited above.

Not every aspect of a key proposal for the spontaneous emergence of dynamical chemical organizations can be scrutinized in a single paper. Here we restricted ourselves to three issues: (i) the probability of the nucleation of reflexively autocatalytic networks, as questioned e.g. by Lifson [26], (ii) the side reaction problem, as raised by Orgel [41] and Szathmáry [28], and (iii) the question of Darwinian evolvability of autocatalytic polymer sets, as left open by the previous investigations by Bagley et al. [14,15]. We think we have advanced promisingly with all three problems in the present work. Our work shows that autocatalytic sets as first devised by Dyson [9,10] and Kauffman [1,11] are theoretically possible despite previous criticisms and, perhaps more interesting, that chemical evolution in these systems can lead to the appearance of viable autocatalytic cores, thus opening the possibility for evolution by natural selection. We have used an abstract chemistry not to avoid real chemistry, but to seek general principles. For example, selection between autocatalytic cores may even be a possibility in combinatorial inorganic systems evolving in iCHELL compartments [46]. Naturally, one cannot be satisfied with abstract toy chemistry for long. But there is always a first step, and the scenario outlined here should give us hope that it is worthwhile to explore the idea further. After all, the pre-template Darwinian dynamics of rare core production and selection described here - fundamentally different from the mechanism advocated by Kauffman [1] and dismissed by Eigen [18] - is the only viable proposal so far for how autocatalytic reaction networks could accumulate adaptations.

## Methods

Following Farmer et al. [12] a simplified model of catalyzed linear polymer reactions was devised consisting of polymers of alphabet size *B *= 2. Reversible, ligation (condensation) and cleavage reactions, catalyzed by another polymer, were modelled. Reactions were of the form:

(2) $$R_{\text{1}}\;+\;R_{\text{2}}\;\overset{C}{↔}\;P_{\text{1}}$$

where *R*_1 _and *R*_2 _are the concentrations of reactants, *P*_1 _is the concentration of the product of a condensation reaction, and *c *is the catalyst. No attempt was made to relate the structure (i.e. sequence) of a polymer to its catalytic or reactive function. Each molecule has a certain probability *P *of catalyzing each theoretically possible ligation-cleavage reaction, and the reactions in which a polymer will participate, along with the catalysts, are determined randomly.

The chemical kinetics approximates the behaviour of catalyzed reactions enclosed in a compartment. We assumed identical binding velocity for all intermediates. Only the catalytic rates vary in magnitude from 10 to 1000. The food set contained polymers up to length *M *= 4. Food was present at initial concentration *F_c _*and added continuously at rate *F*_input_, and all molecular species have a first order decay rate *k*_*d*_. We typically used 10^-5^M as a minimum concentration threshold below which no molecule of the species exists in the reactor (for more details see Additional file 1).

In order to address Lifson's criticism [26], the parameter *P *was replaced by two independent probabilities: the probability *P' *that the molecule is a catalyst, and the probability *P" *that a molecule catalyzes a given reaction. In models including inhibition, when a new molecular species is first produced (in a manner analogous to the determination of catalytic reactions) it is determined which other species in the reactor the new species will inhibit with probability *K*, and which existing species will poison the new species also with probability *K*. The probability of inhibition varied from 0 to 1. In the novel species model uncatalyzed reactions among available molecular species took place with a low probability (the concentration of any molecular species had a probability ranging from 0 to 0.01 to increase over threshold), producing random novel species in low copy number from the shadow.

The three versions of the chemical model (original, inhibition and with novel species), all confined into compartments, were subjected to the same tests in order to assess their evolvability. We approximated the number of attractors in chemical networks by fixing the reaction network and shuffling the chemical concentrations (by choosing random pairs of species and swapping their concentrations) several times in order to sample various initial conditions. After shuffling, the network dynamics are run for 1000 time steps that appeared enough to reach a (new) attractor. Even if multiple attractors exist, stochastic division might not generate sufficient variation to transition between them. To test this, compartments enclosing the generative chemistry were allowed to grow for a fixed period of time after which compartment splitting was modelled by taking a polyhypergeometric distribution of molecular contents. Typically the propagule size was 500 molecules to allow sufficient variability, also permitting loss of species upon division. In addition, a low probability of spontaneous appearance in low copy number of species that already exist in the platonic reaction network (but have gone below threshold concentration) was assumed, allowing the re-emergence of lost species.

In the artificial selection experiment a population of 10 compartments was isolated for a fixed generation period. After this time the fitness of each compartment was assessed and production of the next generation of 10 new compartments occurred by taking 800 molecule propagules from compartments on the basis of fitness proportionate selection; i.e. compartments were chosen to populate the next generation in proportion to their fitness by using roulette-wheel selection with elitism [34]. Elitism was used to always sample at least one propagule from the individual with the highest rank in any given generation. Two different targets of selection were imposed. First, fitness of a compartment was defined as the total mass of non-food species (sum of the concentration multiplied by length for all non-food species) present in a compartment at the end of the growth phase, just prior to division. Second, the fitness was defined as the reciprocal of this value. As the last step, the response of the chemical networks to natural selection was tested in numerical simulations. We introduced a classical Moran process [35] with the following parameters: propagule size was 800 molecules, division occurred at the compartment size (*η*) of approximately half of the equilibrium size (approx. 2.2 mass units of non-food; mass is calculated as concentration multiplied by length) and the population size (N) was set to 100 compartments. Selection for a specific target was implemented by multiplying the rates of all reactions by a selective advantage *S *(set to 1.01 or 1.1) if it matched the characteristic composition of the desired attractor. The lack of core were defined as none of its members present at concentration > 0.02.

## Competing interests

The authors declare that they have no competing interests.

## Authors' contributions

VV carried out the structural analysis of autocatalytic sets and CF performed the simulations. All authors participated in design of the study and writing the manuscript. All authors read and approved the final manuscript.

## Supplementary Material

Additional file 1 **Spontaneous Formation and Evolution of Autocatalytic Sets within Compartments**. This document provides information on methodological details, gives examples of autocatalytic loops, and discusses results that were only briefly mentioned in the main text. Click here for file

## Reviewer's comments

### Reviewer 1: Prof. William Martin

*Reviewer's comments: *This is a thoughtful but narrowly focussed paper, that is possibly too far removed from real chemistry and real metabolism in general design to be suited to BD. Overall, though, I think that the authors have presented a contribution worth publishing. My main complaint would be that they overlook the possibility of prexisting inorganic catalysts that are not part of their reaction products and the possibility that things chemical fall into place more along the lines of thermodynamics than along the lines of mathematics.

*Authors' response: *Indeed, we used chemistry more for inspiration than for the actual derivation of model parameters. The main reason for this narrow focus was that we found the mathematical foundations of selectability in reaction networks utterly confused. How could we study which real chemistry could produce evolvable units without understanding what exactly makes a reaction network selectable? Naturally, our work is just a first step towards a more rigorous study of evolvable networks; and next we must understand the kind of chemistry that could realize distinct autocatalytic cores. We are planning to implement a more realistic rendering of chemistry, one based on affinity dependent on pattern matching, in the near future. It is indeed very important to increase chemical realism in such models, though this needs large computational resources.

*Reviewer's comments: *But the network concept is surely useful here. I cannot see how Oparin fits into this and I am not convinced that this paper really addresses coacervates in any way.

*Authors' response: *The idea of coacervates is not relevant for the paper, only Oparin's once widely accepted, and now widely debated idea of a self-assembling, self-reproducing, compartmentalized proto-metabolism.

*Reviewer's comments: *One misses references to Mike Steel's papers on the mathematics of this topic.

*Authors' response: *This mistake has been corrected.

*Reviewer's comments: *It is excessive to mention Kauffmann's name 20 times in the text, evoking the impression that what was written in the literature is a good deal less important than who wrote it.

*Authors' response: *We agree, and we modified the text accordingly.

*Reviewer's comments: *In the practical sense of actually talking about biochemical origins, the authors seem too concerned about polymerization in my view, they should be more concerned about the source of the monomers. In that respect, papers by Harold Morowitz should be consulted, for example (Morowitz, H. J., J. D. Kostelnik, J. Yang, and G. D. Cody. 2000. The origin of intermediary metabolism. Proc. Natl. Acad. Sci. USA 97:7704-7708),

*Authors' response: *We are less enthusiastic about the Morowitz et al. paper. First, the primitive ancestry of the reductive citric acid cycle has been forcefully advocated by Günter Wächtershäuser before. Second, we still do not have evidence for a non-enzymatically running such cycle (pyruvate carboxylation seems to be an obstacle). Third, we *do *have evidence for a non-enzymatic autocatalytic metabolic cycle: the formose reaction. For the time being we prefer not to be too dogmatic about concrete chemical scenarios. Ultimately it is an experimental issue.

*Reviewer's comments: *as should the chapter:

Amend, J. P., and T. M. McCollom. 2009. Energetics of biomolecule synthesis on early Earth. In "Chemical Evolution II: From the Origins of Life to Modern Society" American Chemical Society, Chapter 4, pp 63-94.

where they will be surprised to find much information that strongly supports portions of their case, if they can bear the thought of what geochemical environment is involved.

*Authors' response: *We are grateful for the reviewer for pointing out the chapter to us. It is now referenced in the article. It is indeed reassuring to see that the Gibbs free energy for the synthesis of amino acids is very favourable at natural sites like the Lost City. We are fully aware that geochemical realism must ultimately be an integral part of the whole story. We have taken the path of classical theoretical biology: *if *certain assumptions are made, what are the *consequences *for evolutionary dynamics. Ever since the work of Manfred Eigen in 1971, students of the origin of life must bear the thought of what selective forces could have been involved.

### Reviewer 2: Dr. Eugene V. Koonin

This article addresses a question of extreme interest and importance, in a way the central question in the origin of life field: is adaptive evolution possible without replicating template polymers, in autocatalytic networks of chemical reactions? A compelling positive answer to this question will effectively be a solution (at least in outline) to the origin of life problem, i.e. a plausible path from a mix of simple organic molecules to a replicating system of modern type. Incidentally, this solution will also imply that there was no RNA World at the beginning of life. The question is by no means new, it has been addressed by many, starting apparently with Oparin who realized the problem clearly, even if only at the verbal level, and including some of the authors of this article who concluded in previous work that autocatalytic networks in general lack the evolvability potential. The outcome of the model and simulations described in the present article is different: the authors conclude that evolvability is characteristic of autocatalytic chemical networks if they have a particular structure and exist in networks of compartments. The new principle that emerges in this work is that of the separation of chemical networks into core and periphery. The small stable core consists of multiple autocatalytic loops whereas the periphery includes non-autocatalytic reactions and is generated by the core. In a sense, the core is analogous to the genotype whereas the periphery is an analog of the phenotype. In the simulations presented in the paper, this system displays bona fide Darwinian selection as well as drift: new cores emerge by 'mutation' and elements of cores can be stochastically lost.

I have no reasons to question the validity of the results within the model and the chosen parameters. The limitations of the work that the authors fully realize are that: i) the model is purely abstract and does not include any real chemistry, ii) even if one accepts the possibility of replication of autocatalytic networks, no path to template-based replication is provided let alone the key genotype-phenotype link via translation. I would add that to me, the central problem is the stability of such a putative non-templated system of replicators in real time: is it all sufficient to provide the springboard for evolution leading to modern-type life forms? There is currently no answer to this key question, and in my opinion, such an answer cannot be reached at the level of abstraction adopted in the present work. However, the authors of the paper notice absolutely correctly that all problems in such a broad and extremely challenging field as the origin of life cannot be possibly addressed in a single paper. In my opinion, this stimulating paper is an important step in a most important direction.

*Authors' response: *An understanding of the kinds of chemical organization that could sustain heredity is logically anterior to the problem of the stability of such organizations. We are planning to investigate the selective origin of template replication within such a framework in the near future. We also think that the origin of the genetic code is a considerably later step (as advocated by one of the authors): an interim (albeit dirty) RNA world must have prevailed, simply as a consequence of the opportunistic nature of evolution by natural selection.

### Reviewer 3: Dr. Anthony Poole

This reviewer provided no comments for publication.
