# Supplementary material for: Evolution before genes
Source: Biol Direct. 2012 Jan 5;7:1. doi: 10.1186/1745-6150-7-1 (PMC3284417; doi:10.1186/1745-6150-7-1)
Supplement: Additional file 1 — Spontaneous Formation and Evolution of Autocatalytic Sets within Compartments. This document provides information on methodological details, gives examples of autocatalytic loops, and discusses results that were only briefly mentioned in the main text. [file 1745-6150-7-1-S1.DOC]

# Additional file 1

# Spontaneous Formation and Evolution of Autocatalytic Sets within Compartments

## Chrisantha Fernando, Vera Vasas, Mauro Santos, Stuart Kauffman, and Eörs Szathmáry

Contents

A $-$ Autocatalytic sets 2

A1. Definitions of autocatalytic sets 2

A2. Structure of autocatalytic sets in Kauffman’s polymer chemistry 2

B $-$ Methods 6

B1. Reimplementation of Farmer et al’s model 6

B2. Chemical network analysis algorithms 12

C $-$ Example of networks with novel viable loops 14

D $-$ Further results on evolvability 19

D1. Alternative attractors 19

D2. Artificial selection 22

References 24

**A $-$ Autocatalytic sets**

**A1 Definitions of autocatalytic sets**

The essential feature of so-called autocatalytic sets is that they can collectively self-replicate (grow exponentially), even if none of their component molecular species can individually self-replicate [1]. It is necessary to distinguish the various $-$sometimes contradictory$-$ definitions of what an autocatalytic set is. Kauffman [2] defines an autocatalytic set as a set of molecules in which “every member of the autocatalytic set has at least one of the possible last steps in its formation catalyzed by some member of the set, and that connected sequences of catalyzed reactions lead from the maintained ‘food set’ to all members of the autocatalytic set”.[[1]](#footnote-2) This is more formally defined by Hordijk and Steel [4], who state that an *R* (sub)set of reactions is called (*i*) reflexively autocatalytic (RA) if every reaction in *R* is catalyzed by at least one molecule involved in any of the reactions in *R*, (*ii*) food-generated (*F*) if every reactant in *R* can be constructed from a small “food set” by successive applications of reactions from *R*, and (*iii*) reflexively autocatalytic and food-generated (*RAF*) if both *RA* and *F*. Note that Kauffman’s definition [2] excluded autocatalytic sets that could not be constructed from the food set and thus corresponds to a *RAF*. Other work has defined autocatalytic sets more abstractly in terms of the catalytic graph: “an autocatalytic set is a graph, each of whose nodes has at least one incoming link from a node belonging to the same graph” [5], analogous to an *RA* set. In a catalytic graph, nodes depict molecules and edges are catalytic actions.

**A2 Structure of autocatalytic sets in Kauffman’s polymer chemistry**

Kauffman’s [2] original mathematical model assumes the following: (*i*) there exists a large “food set” of abundant polymers naturally formed in the environment, up to some low level of complexity; i.e. up to length *M* consisting of *B* types of monomers; (*ii*) each molecule has a certain probability *P* of catalyzing each ligation-cleavage reaction. The model assumes infinite specificity, in other words, a molecule either does or does not catalyze a particular reaction without quantitative variation in efficiency. It was demonstrated that above a certain catalytic probability threshold a chain reaction is triggered and due to catalytic closure autocatalytic sets appear.[[2]](#footnote-3)

Hordijk and Steel [4] verified this claim by generating random networks of reversible ligation/cleavage reactions between strings up to length *n* = 20, where each molecule had the probability *P* of catalyzing each reaction. At low values of *P* they found unconnected *RAF*s utilizing separate food sets, but at higher values a percolation phenomenon produced a fully connected *RAF* sets[[3]](#footnote-4).

Our results after implementing Farmer et al. [7] model (see section B $-$ Methods) are in agreement with those of Hordijk and Steel [4]. With small *P* the number of molecular species is small compared to the size of the food set and they form jets with acyclic catalytic graphs. Catalytic closure in reached when all food species are catalyzed (non-food species can only appear if catalyzed) and the catalytic graph consists of one strongly connected component (core) and its periphery (Figure S1). However, autocatalytic cores consist mainly of suicidal autocatalysts rather than viable autocatalytic loops (Figure S2).


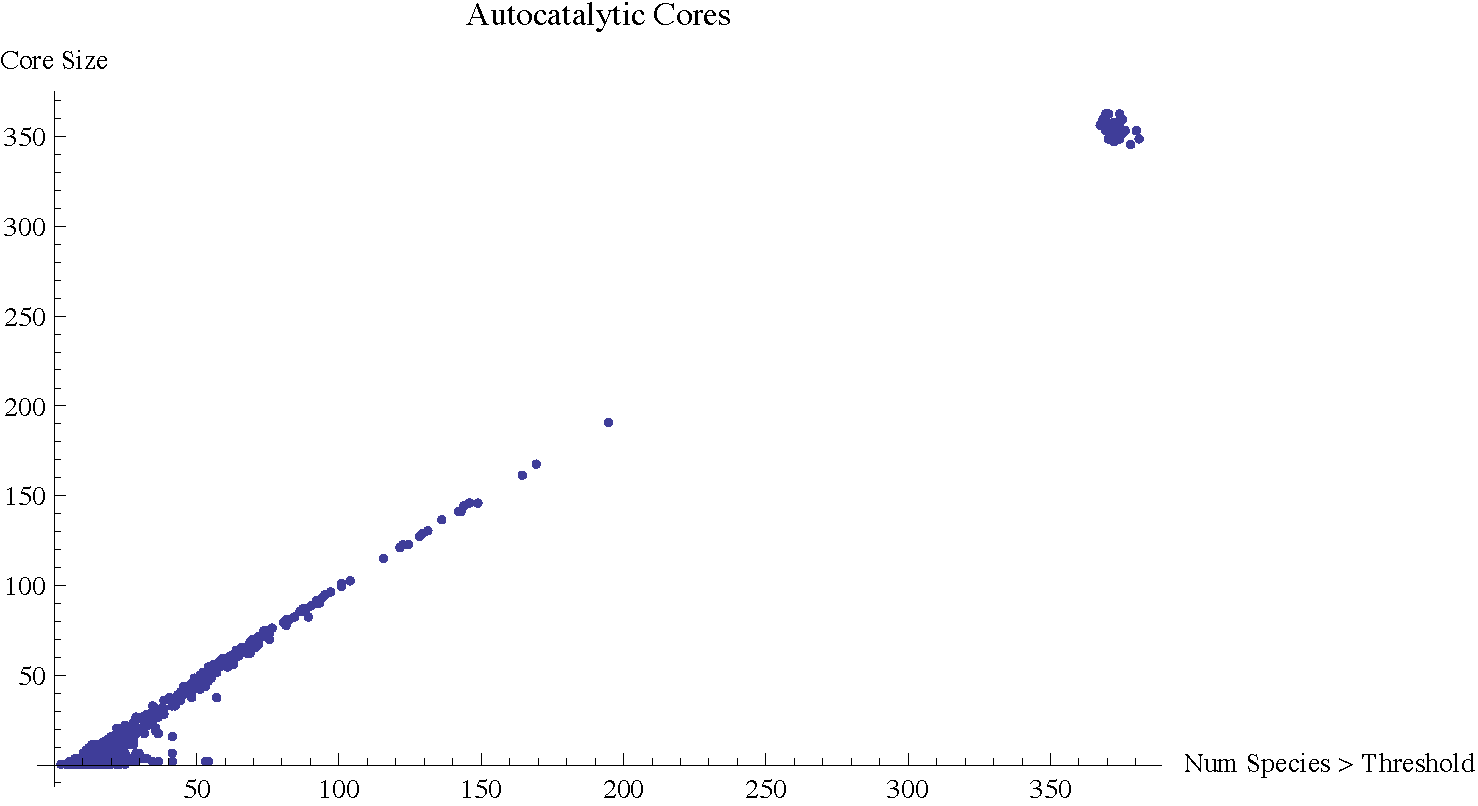


Figure S1. Distribution of sizes of autocatalytic cores in Farmer et al. [7] model. For large networks most species maintained above threshold are in one autocatalytic core (strongly connected component in the catalytic graph). Each dot corresponds to a different chemical network generated with *P* = 0.00022.


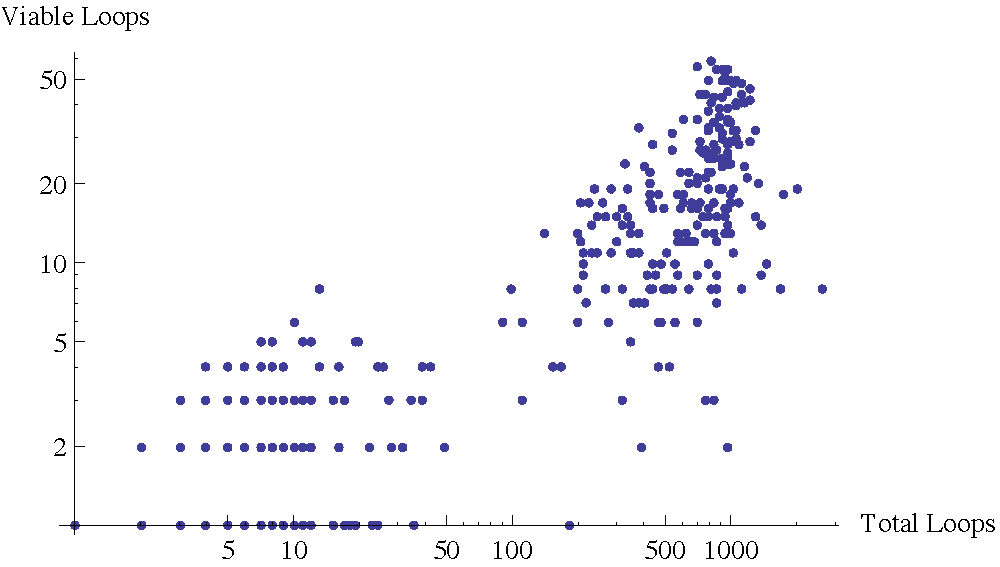


Figure S2. Frequency of viable loops in Farmer et al. [7] model. Only a minority of catalytic loops are viable. Each dot corresponds to a different chemical network generated with *P* = 0.00022.

**B $-$ Methods**

**B1 Reimplementation of Farmer et al.’s model**

In Farmer et al. [7] a simplified model of catalyzed linear polymer reactions was devised consisting of polymers of alphabet size *B* = 2. Reversible, ligation (condensation) and cleavage reactions were permitted. Only reactions catalyzed by another polymer were modeled. Spontaneous stochastic production of uncatalyzed species whilst ignored in the model would be expected to occur in a realistic reactor (see below). Reactions were of the form:

[1]

where $R_{1}$ and $R_{2}$ are the concentrations of reactants, $P_{1}$ is the concentration of the product of a condensation reaction, and *c* is the catalyst. Cleavage involves $P_{1}$ breaking down into $R_{1}$ and $R_{2}$, the bi-directional arrow indicating reversibility. There is no attempt to relate the structure (i.e. sequence) of a polymer to its catalytic or reactive function. The reactions in which a polymer will participate are determined randomly. The catalyst for the reaction is also chosen randomly. From the initial food set (each member of which is present at initial concentration $F_{c}$ and added continuously at rate $F_{\text{input}}$) the maximum number of possible distinct condensation and cleavage reactions that could theoretically occur is determined. Because at the outset, all species below and including length *M* are present, cleavage reactions within the food set are not modeled. The food set species are placed into the inner ring of species, $S_{I}$.

The number of new reactions to be generated is determined by multiplying the number of possible reactions by probability *P*. The number of possible reactions is a function of the numbers of species in the inner ($S_{I}$) and outer ($S_{O}$) rings that are present above the concentration threshold, *T*. The method for generating these rings is described below. Note: it would have been more realistic to determine the number of reactions using a Binomial distribution with probability of success per possible reaction equal to *P*, and where the sample size *n* equals the population size (number of possible reasons) *N*. If a novel species is formed in a new reaction then it is placed into the outer ring of species, S_O_. The reaction kinetics are then simulated for long enough for them to reach steady-state for that particular reaction network.

The main loop of the simulation is then entered. This involves again calculating all new reactions between the newly produced species in $S_{O}$ and the old species in $S_{I}$, and placing new species in a temporary ring, $S_{T}$. The outer ring species are then moved to the inner ring, and the temporary species are moved to the outer ring. Then the dynamics are run for one hundred time-steps. If this results in a species in the inner ring obtaining a concentration greater than threshold *T* (for the first time) then that species is moved to the outer loop where it can take part in the next round of novel reaction calculations (and its candidate status is updated to active). This simulates a minimum concentration below which no molecule of the species exists in the reactor. A concentration of 10^-9^M corresponds to a single atom in a compartment the size of a bacterium. Higher thresholds have to be used in simulations, due to the difficulty of simulating large reaction networks, e.g. we typically use 10^-5^M as a concentration threshold. This loop repeats until the simulation is terminated if there are too many species or too many condensation reactions to simulate, or if the maximum simulation time is reached.

The chemical kinetics approximates the behavior of catalyzed reactions, assuming identical binding velocity for all intermediates. Only the catalytic rates, $v_{o}$*,* vary in magnitude from 10 to 1000. The concentration of each species is separated into a bound, $\left[S\right]_{b}$, and a free part, $\left[S\right]_{f}$. The bound concentration represents the sum of all concentrations of intermediates in which *S* participates. The forward rate of a condensation reaction (e.g. Eq. 1) is given by

$v_{f}=v_{o}k_{f}\left[C\right]_{f}\left[R_{1}\right]_{f}\left[R_{2}\right]_{f}$ [2]

and the backward rate by

$v_{b}=v_{o}k_{b}\left[C\right]_{f}\left[P_{1}\right]_{f}$ [3]

where $k_{f}$and $k_{b}$ are the forward and backward equilibrium constants, respectively. The change in free concentration of the species due to reactions are given by the reaction terms

$\frac{\left[R_{1}\right]_{f}}{dt}=-v_{f}$ [4.1]

$\frac{\left[R_{2}\right]_{f}}{dt}=-v_{f}$ [4.2]

$\frac{\left[P_{1}\right]_{f}}{dt}=-v_{b}$ [4.3]

$\frac{\left[C\right]_{f}}{dt}=-v_{b}-v_{f}$ [4.4]

When addition of the food set, formation of bound intermediates, and decay of species is considered, the total change of concentration of a species is given by

$\frac{\left[S\right]_{f}}{dt}=\sum\left[reactions\right]+k_{u}\left[S\right]_{b}-k_{d}\left[S\right]_{f}+F_{input}H\left(M-L\right)$ [5

]

where $k_{u}$is the unbinding velocity or dissociation constant and $k_{d}$is the decay rate. The last term is the Heaviside function $H\left(x\right)=1$ for $x>0$, $H\left(x\right)=0$ for $x<0$; *L* is the length of species *S*; and $F_{input}$ is the rate of food input. The change in bound concentrations due to a reaction are given by

$\frac{\left[R_{1}\right]_{b}}{dt}=v_{b}$ [6.1]

$\frac{\left[R_{2}\right]_{b}}{dt}=v_{b}$ [6.2]

$\frac{\left[P_{1}\right]_{b}}{dt}=v_{f}$ [6.3]

$\frac{\left[C\right]_{b}}{dt}=v_{b}+v_{f}$ [6.4]

and the corresponding total change of concentration for bound species is

$\frac{\left[S\right]_{b}}{dt}=\sum\left[reactions\right]-k_{u}\left[S\right]_{b}-k_{d}\left[S\right]_{b}$ [7]

The full details of the algorithm and parameters used are described in Table 1 and Algorithm 1.

Table 1. Parameters, initial values and variables used in Farmer et al. [7].

| Parameter | Symbol | Value/Range |
| --- | --- | --- |
| Alphabet Size | *B* | 2 |
| Firing Disk Radius | *M* | 4 |
| Probability of Catalysis | *P* | 0.0032 - 0.00028 |
| Concentration Threshold | *T* | 0.00001 |
| Time step | τ | 0.0001 |
| Iterations until steady-state | Φ | 100 |
| Catalytic velocity per catalyst | *v_o_* | 10 < v_o_ < 1000 |
| Condensation rate constant | *k_f_* | 1 |
| Cleavage rate constant | *k_b_* | 10 |
| Decay rate constant | *k_d_* | 1.0 |
| Dissociation rate constant | *k_u_* | 1000 |
| Initial Value |  |  |
| Initial firing disk species concentration | *F_c_* | 2.0 |
| Food set input rate | *F_input_* | 2.0 |
| Variables/States |  |  |
| Species | *S* |  |
| Species in inner disk | *S_I_* |  |
| Species in outer disk | *S_O_* |  |
| Species in temporary disk | *S_T_* |  |
| Species Length | *S_L_* | 1 < *S_L_* < ∞ |
| Species concentration | [*S*] | 0 < [*S*] < ∞ |
| Species concentration (free) | [*S*]*_f_* | 0 < [*S*] < ∞ |
| Species concentration (bound) | [*S*]_b_ | 0 < [*S*] < ∞ |
| Species catalyses a reaction | *S_cat_* | 1 / 0 |
| Species candidate status | *S_cand_* | 1 / 0 |
| Reactant x / Product x | *R_x_* / *P_x_*­ |  |

Algorithm 1: An interpretation of the algorithm described in Farmer et al. [7].

1. Initialize firing disk

Create all firing disk polymers <= M in length, at initial concentration F_c_.

2. Create new condensation reactions

Number of condensation reactions in firing disk (R_cond_) = *P*.*c.S_I_^2^*

*c* = num of possible catalysts in inner disk, i.e. the subset of S_I_ satisfying conditions: [S_I_] > T, S_cat_ = 0.

*For R_cond_*

Choose two random species satisfying conditions: [S_I_] > T.

Create random condensate at concentration 0, S_cand_ = 1[[4]](#footnote-5), catalysed by a random species satisfying conditions: [S_I_] > T, S_cat_ = 0. Set S_cat_ = 1

Assign new condensate species to outer disk S_o_

*End For*

3. Run dynamics for Φ steps with time-step τ

Reset all temporary concentrations

*For each condensation reaction R_1_+ R_2_ 🡪 P_1_ where [C] > T & ( [R_1_] & [R_2_] > T || [P_1_] > T)*

*For each catalyst j that catalyses this reaction*

*End For*

*End For*

*For each cleavage reaction R_1_ 🡪 P_1_+ P_2_ , where [C] > T & ( [P_1_] & [P_2_] > T || [R_1_] > T)*

*For each catalyst j that catalyses this reaction*

*End For*

*End For.*

Adjust distribution between bound and unbound species

*For all Species*

If (S_L_ <= M) then *[S]f_temp* =  *[S]f_temp +* .F_input_

*End For*

[S]_b/f_ += [S]b_temp/f_temp

4. MAIN LOOP

*For N iterations or until Stop = 1*

4.1. Assign condensations between one old and one new species, and between two new species, catalysed by old or new species.

Num new condensations = S_O_^2^ + 2S_I_S_O_ for all [S] > T

Num possible catalysts = S_I_ + S_O_ , where S_cand_ = 0

Num reactions = P x Num possible catalysts x Num new condensations

Create reactions: S_cat_ = 1 for the catalysts used. Put New species in S_temp_.

4.2. Assign cleavages of new species, catalysed by old or new species.

Num new cleavages = Total number of bonds in S_O_

Num possible catalysts = S_I_ + S_O_ , where S_cand_ = 0

Num reactions = P x Num possible catalysts x Num new condensations

Create reactions: S_cat_ = 1 for the catalysts used. Put New species in S_temp._.

4.3. Assign condensations of old species, catalysed by new species.

Number reactions = P x S_O_ S_I_ ^2^

Create reactions: S_cat_ = 1 for the catalysts used. Put New species in temp store.

4.4. Assign cleavages of old species, catalysed by new species.

Num Reactions = P x S_O_ x Number of bonds in S_I_

Create reactions: S_cat_ = 1 for the catalysts used. Put New species in S_temp_.

4.5. Move S_O_ to S_I_ and move S_temp_ to S_O_.

4.6. Run step (as in 3).

4.7. If [S_I_] > T for the first time: S_cand_ = 0, move to S_O_

*End For*

**B2 Chemical network analysis algorithms**

The first part of the algorithm for characterizing the structure of the catalytic reaction network obtained is to determine all the strongly connected components of the catalytic reaction graph. In a catalytic graph, nodes depict molecules and edges are catalytic actions. The strongly connected components (SCCs) correspond to the viable cores. SCCs were detected using a standard function from the *Boost* graph library [8].

The next step was to detect all the directed cycles in the catalytic graph. Cycles can only exist within SCCs. Detection of cycles is achieved using a distributed salesman algorithm on a catalytic graph. The catalytic graph is first modified by giving a number to each chemical type (node). Then the following algorithm is undertaken to detect all directed cycles.

Algorithm 2: Detecting cycles in a catalytic graph .

1. For each strongly connected component
2. Choose a random node in the SCC and place a new salesman on it
3. Number this salesman according to the number of the node
4. Place a pointer to the node into its visitedList
5. Place the salesmen in the oldSalesman list
6. While (oldSalesman list is not empty)
7. Make empty newSalesman list
8. For each salesman S in oldSalesman list
9. For each reachable node X from the node of salesman S
10. If(If reachable node number X >= S number)
11. Make new salesman S’ with same number

12. and visitedList as parent S

13. Add pointer to node X to S’ visitedList

14. If(cycle detected in visitedList)

15. If cycle is new store in cycleList

16. Kill Salesman S’

17. delete oldSalesman List

18. copy newSalesman List to oldSalesman list

Once all the possible cycles have been detected in the catalytic graph it is necessary to determine the viability of each cycle. For a cycle to be viable, it must be possible to obtain from the food set a minimal subset of the substrates from which the members of the cycle can be catalyzed by other members of the cycle. Only if this is the case is there the potential for autocatalysis, i.e. for exponential growth of the cycle. The algorithm for detecting viable cycles is shown below.

Algorithm 3: Identifying viable cycles.

1. For each cycle
2. Initialize extendedFoodSet with the food set
3. Grow extendedFoodSet with species created from reactions of species in extendedFoodSet

whose product is not a loop member

1. For each member of the cycle
2. Determine all reactions in which this member is produced as a product
3. If at least one of the necessary set of reactants for production of this

member is in the extendedFoodSet, mark this member as validly producible.

1. If all members of the cycle are validly producible, then mark cycle as valid.

**C $-$ Examples of networks with novel viable loops**

***Example 1*** *(used in main text)*

*Reaction network prior to spontaneous reactions*

abaa + aaab - aab -> abaaaaab
abbb + abbb - abaa -> abbbabbb
abbb + babb - baba -> abbbbabb
aabb + bba - aa -> aabbbba
baaa + aaba - a -> baaaaaba
abbbabbb + b - aaaa -> abbbabbbb
aabbbba + bbab - baa -> aabbbbabbab
baa + aaba - abbbbabb -> baaaaba
abbbabbbb + abbbbabb - aaba -> abbbabbbbabbbbabb
abbbabbbb + bab - bb -> abbbabbbbbab
bbba + baba - abbbabbbb -> bbbababa
bbbababa + baba - aaab -> bbbababababa
bbbababa + abbbabbb - ab -> bbbababaabbbabbb
bbbababababa + aa - bbbababa -> bbbababababaaa
bbbababaabbbabbb + ab - bba -> bbbababaabbbabbbab
bbbababababaaa + bbb - bab -> bbbababababaaabbb
bbbababaabbbabbbab + abaaaaab - babb -> bbbababaabbbabbbababaaaaab
bbbababababaaabbb + aba - bab -> bbbababababaaabbbaba
abaa + bbbababaabbbabbb - bbbababababaaabbb -> abaabbbababaabbbabbb

*New reactions incorporated after a spontaneous species appears*

abaa + aaab - aab -> abaaaaab
abbb + abbb - abaa -> abbbabbb
abbb + babb - baba -> abbbbabb
aabb + bba - aa -> aabbbba
baaa + aaba - a -> baaaaaba
abbbabbb + b - aaaa -> abbbabbbb
aabbbba + bbab - baa -> aabbbbabbab
baa + aaba - abbbbabb -> baaaaba
abbbabbbb + abbbbabb - aaba -> abbbabbbbabbbbabb
abbbabbbb + bab - bb -> abbbabbbbbab
bbba + baba - abbbabbbb -> bbbababa
bbbababa + baba - aaab -> bbbababababa
bbbababa + abbbabbb - ab -> bbbababaabbbabbb
bbbababababa + aa - bbbababa -> bbbababababaaa
bbbababaabbbabbb + ab - bba -> bbbababaabbbabbbab
bbbababababaaa + bbb - bab -> bbbababababaaabbb
bbbababaabbbabbbab + abaaaaab - babb -> bbbababaabbbabbbababaaaaab
bbbababababaaabbb + aba - bab -> bbbababababaaabbbaba
abaa + bbbababaabbbabbb - bbbababababaaabbb -> abaabbbababaabbbabbb
bbaa + bbbababababa - baaaa -> bbaabbbababababa
bbaabbbababababa + baaaaba - abbbabbbb -> bbaabbbabababababaaaaba
b + aaaa - bbaabbbababababa -> baaaa
aabb + aaa - abbbbaa -> aabbaaa
aabbaaa + bab - baaa -> aabbaaabab
b + abbb - aabbaaa -> babbb
aaa + abaa - babbb -> aaaabaa
abaaaaab + abaa - aaaabaa -> abaaaaababaa
abab + ba - aabbaaabab -> ababba
abb + bbaa - abaaaaababaa -> abbbbaa

*New cores*

bbaabbbababababa  -> baaaa  -> bbaabbbababababa
abbbbaa  -> aabbaaa  -> babbb  -> aaaabaa  -> abaaaaababaa -> abbbbaa

***Example 2***

*Reaction network prior to spontaneous reactions*

abaa + bab - abbb -> abaabab

aba + aaba - aab -> abaaaba

aa + aaaa - aba -> aaaaaa

aabb + baa - bbba -> aabbbaa

aabb + aab - abbb -> aabbaab

aabbbaa + aabbbaa - aaaaaa -> aabbbaaaabbbaa

abaabab + b - abab -> abaababb

aaaa + ab - aaaaaa -> aaaaab

aabbbaaaabbbaa + abb - bbbb -> aabbbaaaabbbaaabb

abaababb + aaaaaa - b -> abaababbaaaaaa

aaa + bab - abaababb -> aaabab

aaabab + abba - aaa -> aaabababba

abaababbaaaaaa + a - aaabab -> abaababbaaaaaaa

aaabababba + bab - bbab -> aaabababbabab

aaabababbabab + abb - bbba -> aaabababbabababb

aaabababbabababb + bbbb - aaabababbabab -> aaabababbabababbbbbb

*New reactions incorporated after a spontaneous species appears*

babb + baab - aaaaa -> babbbaab

babbbaab + babbbaab - aaab -> babbbaabbabbbaab

aaaaa + b - babbbaab -> aaaaab

babbbaabbabbbaab + aaaaa - a -> babbbaabbabbbaabaaaaa

babbbaabbabbbaabaaaaa + abaababbaaaaaa - aba ->

babbbaabbabbbaabaaaaaabaababbaaaaaa

*New core*

aaaaa -> babbbaab -> aaaaa

***Example 3***

*Reaction network prior to spontaneous reactions*

b + aa - aaaa -> baa

aaa + aaab - bab -> aaaaaab

abaa + bbab - aaab -> abaabbab

aba + abaa - bba -> abaabaa

abaabbab + aaba - bbba -> abaabbabaaba

ab + baaa - abaabaa -> abbaaa

abbaaa + abb - bbb -> abbaaaabb

*New reactions incorporated after a spontaneous species appears*

aaaaa + ab - aaaaa -> aaaaaab

*New core*

aaaaa -> aaaaa

***Example 4***

*Reaction network prior to spontaneous reactions*

a + aaaa - baa -> aaaaa

baa + baaa - bbaa -> baabaaa

bbb + aba - bbb -> bbbaba

aaaa + baaa - bbbb -> aaaabaaa

bbbaba + bbaa - ab -> bbbababbaa

aaaaa + bb - b -> aaaaabb

aba + a - aaaabaaa -> abaa

aaaaabb + aaaaa - aabb -> aaaaabbaaaaa

bba + aaaa - bbbababbaa -> bbaaaaa

bbaaaaa + aaab - bba -> bbaaaaaaaab

bbba + bb - aaaaabbaaaaa -> bbbabb

bbaaaaaaaab + aaaa - bbbaba -> bbaaaaaaaabaaaa

abb + abaa - bbaaaaaaaab -> abbabaa

abbabaa + abaa - bbba -> abbabaaabaa

abbabaaabaa + aaaaabbaaaaa - bbbababbaa -> abbabaaabaaaaaaabbaaaaa

bbaaaaaaaabaaaa + aaaaabb - ba -> bbaaaaaaaabaaaaaaaaabb

*New reactions incorporated after a spontaneous species appears*

aaa + aaba - bababbbaaaaaab -> aaaaaba

aaaaaba + babb - bbaaaaaaaab -> aaaaabababb

abaa + bba - aaaaabababb -> abaabba

abaabba + bab - abb -> abaabbabab

baba + bbba - abaabba -> bababbba

bababbba + aaaa - bb -> bababbbaaaaa

bbba + aab - bababbba -> bbbaaab

bbbaaab + bbb - ab -> bbbaaabbbb

bababbbaaaaa + ab - bbbaaabbbb -> bababbbaaaaaab

aaaaabbaaaaa + aaaa - bbbaaabbbb -> aaaaabbaaaaaaaaa

bbbaba + aab - abaabbabab -> bbbabaaab

bbbabaaab + ab - aaa -> bbbabaaabab

bbaaaaaaaab + a - bbbabaaab -> bbaaaaaaaaba

bbbabaaabab + bbbaaabbbb - abaabbabab -> bbbabaaababbbbaaabbbb

bbaaaaaaaaba + baba - abaabbabab -> bbaaaaaaaabababa

bbaaaaaaaabababa + bbbaba - abab -> bbaaaaaaaababababbbaba

aabb + aaaa - bbaaaaaaaabababa -> aabbaaaa

aabbaaaa + aaba - aaba -> aabbaaaaaaba

aaaaa + aaab - aabbaaaa -> aaaaaaaab

aaaaaaaab + abaabba - aaba -> aaaaaaaababaabba

aaaa + bbbabb - aaaaaaaab -> aaaabbbabb

aaaaa + bbbababbaa - aabbaaaaaaba -> aaaaabbbababbaa

aaaabbbabb + aabb - aaaaabbaaaaa -> aaaabbbabbaabb

aaaabbbabb + aaaaabb - bbbabb -> aaaabbbabbaaaaabb

abaa + bbaaaaaaaaba - aaaabbbabb -> abaabbaaaaaaaaba

*New core*

bababbbaaaaaab -> aaaaaba

aaaaaba -> aaaaabababb

aaaaabababb -> abaabba->

abaabba-> bababbba

bababbba -> bababbbaaaaa, bbbaaab

bbbaaab -> bbbaaabbbb

bababbbaaaaa, bbbaaabbbb -> bababbbaaaaaab


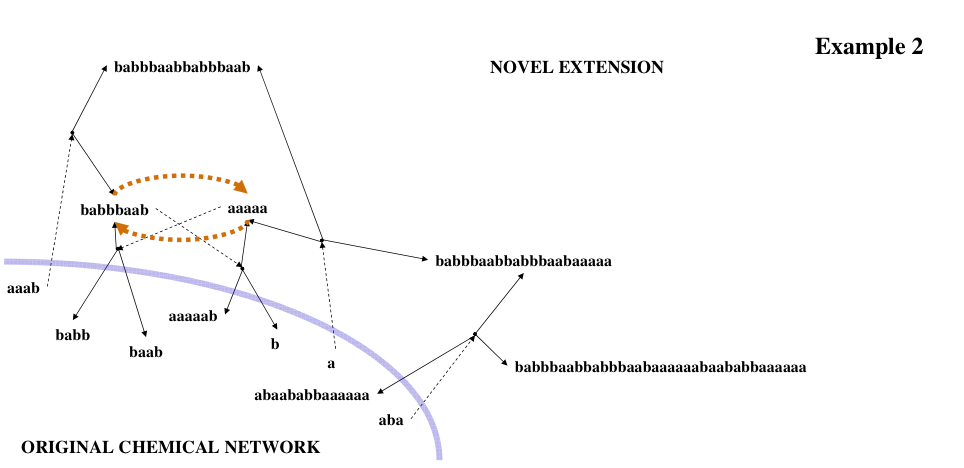


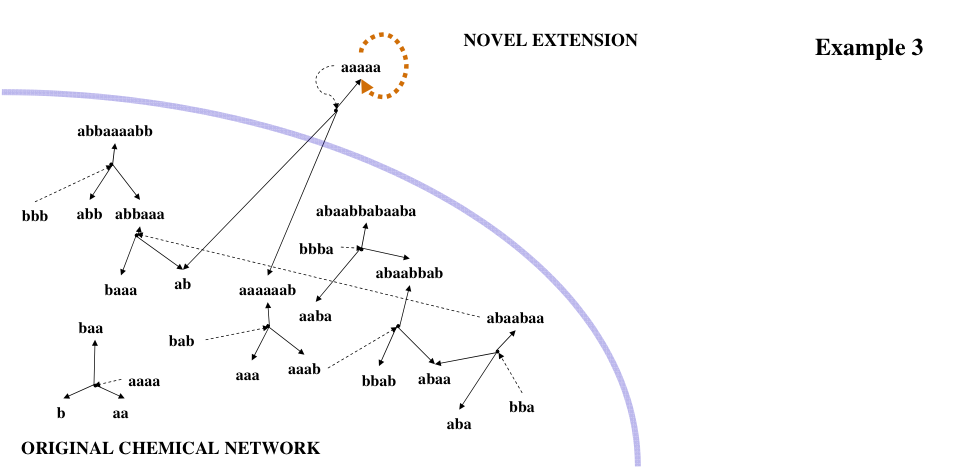


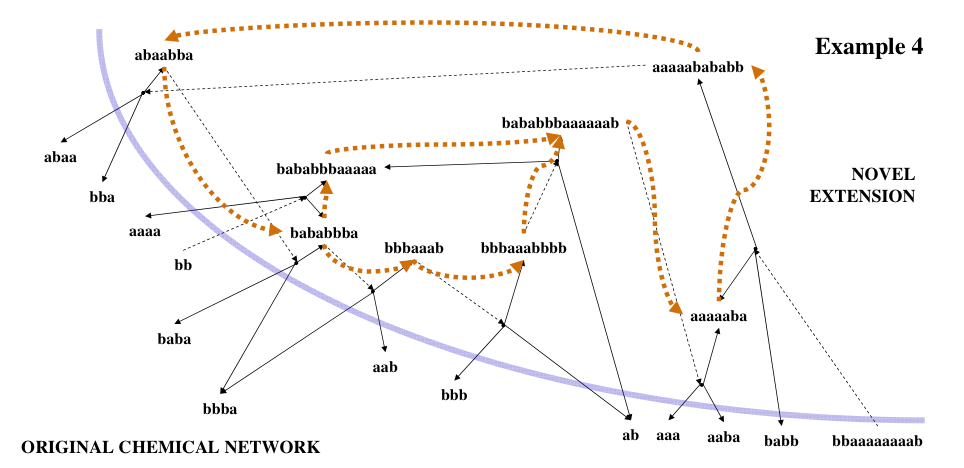


Figure S3. Examples of viable loop organization. Solid lines: reactions; dotted lines: catalytic activities. Orange dotted lines show the superimposed selfreproducing loop. The original network is not shown in detail.

**D $-$ Further results on evolvability**

**D1 Alternative attractors**

The original Farmer-type networks of catalyzed cleavage and ligation reactions (generated with *P* = 0.00022) have only one attractor; i.e. are not capable of variation and cannot be a basis for pre-template based heredity. Networks with inhibition (*P* = 0.00024, *K* = 0.005) exhibit multiple attractors. In the five cases of networks in which the addition of spontaneous new species resulted in the ignition of a novel viable loop, there were multiple attractors in each case found after random shuffling of concentrations (Figure S4).

Networks with inhibition are only very rarely able to spontaneously transit between attractors, and even then they transit periodically or chaotically. On the other hand, spontaneous transitions occur between attractors of the networks that have non-food generated viable loops that were ignited by spontaneously produced shadow species. In both types of network the separate attractors are of different stabilities. Most of the time is spent in one attractor, with only occasional generations spent in the less stable attractor (Figure S5).


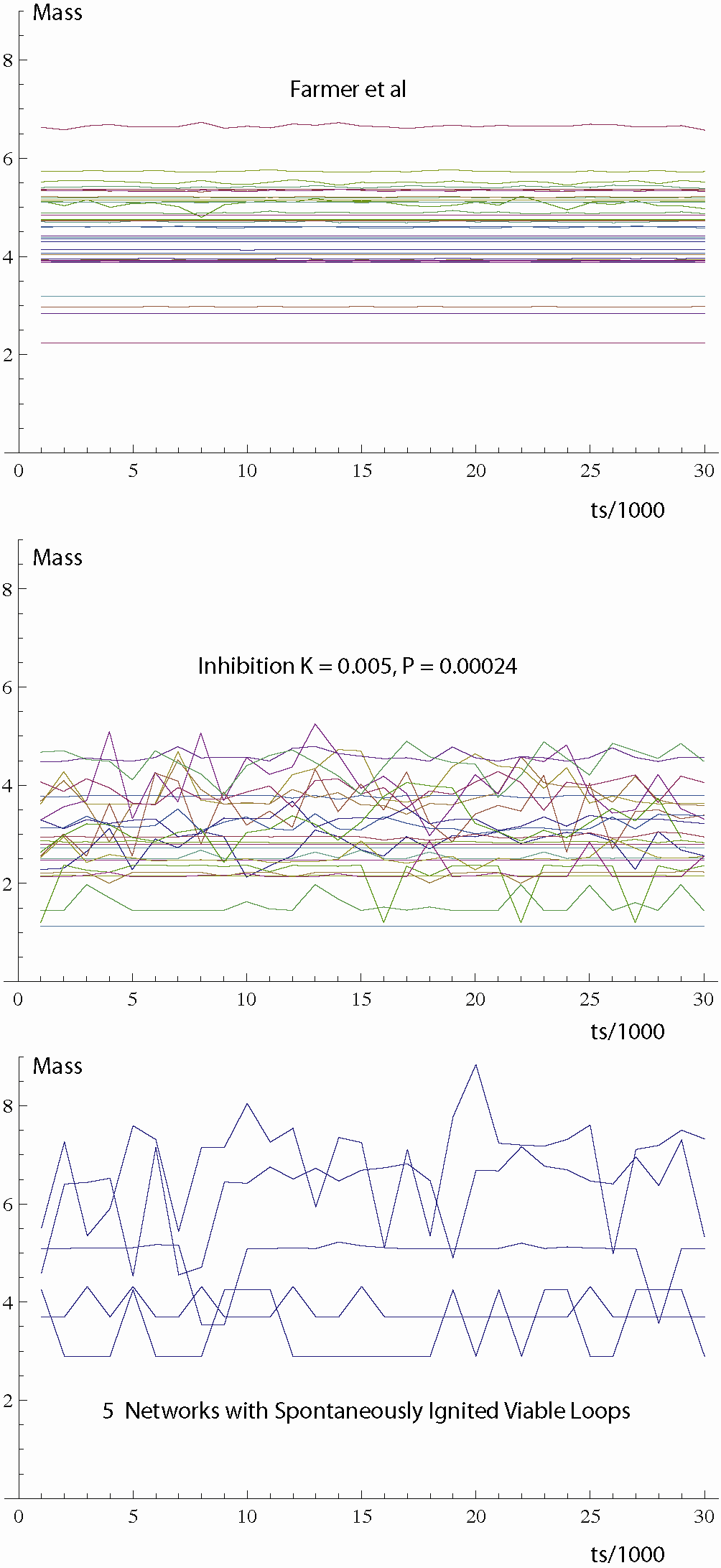


Figure S4. Searching for different attractors. Each line shows a different run where the concentrations of molecular species were shuffled every 1000 time steps for the same chemical network. (Top) In Farmer et al.’s [7] original model, there are no major changes in mass after shuffled concentrations. (Middle) However, where there is inhibition, shuffling results in a different mass 1000ts after shuffling, indicating the presence of a distinct attractor. (Bottom) In the five cases where a network was produced that had increased mass due to incorporation of a novel viable loops, there are always clearly distinct (typically two) attractors that the system settles into.


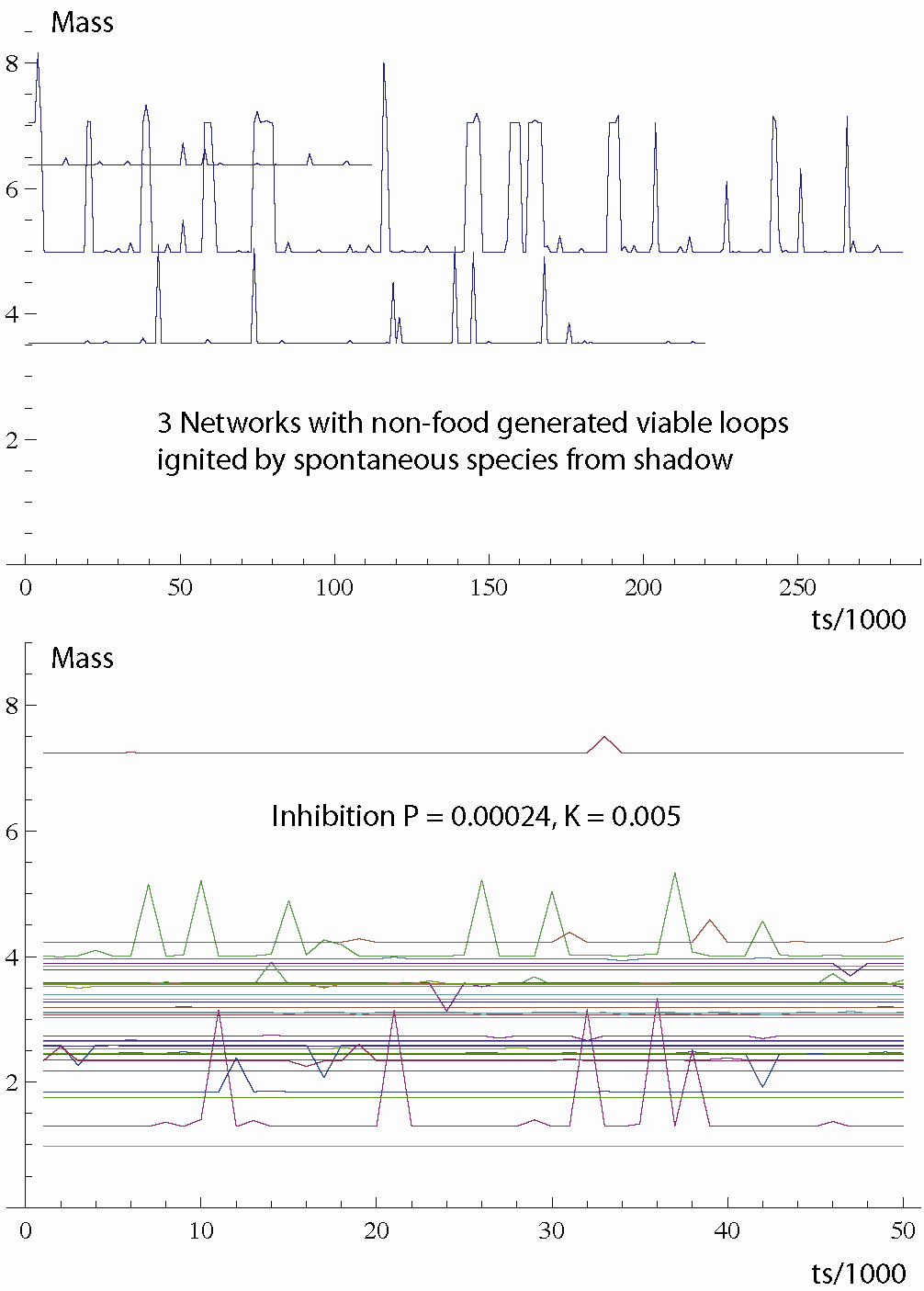


Figure S5. Spontaneous transitions between attractors. Growth and division of compartments result in transitions between existing attractors due to sampling noise during division and spontaneous reappearance of lost molecular species. (Top) Networks with non-food generated viable loops are typically able to transit between attractors. (Bottom) Networks with inhibition are only rarely able to spontaneously transit between attractors.

**D2 Artificial selection**

We applied artificial selection to a population of replicating individuals to test the selectability of alternative attractors. Networks with inhibition could not be stable selected (Figure S6). On the other hand, reaction networks that had obtained a novel viable loop ignited by a spontaneous appearance of a shadow species responded to selection. In the absence of selection the population fluctuated more or less randomly between the two attractors (Figure S7 bottom). However, when artificial selection was imposed the population moved in the expected direction; that is, towards the high mass attractor when total mass of non-food species was defined as the fitness criterion (Figure S7 top), or toward the low mass attractor when fitness was defined as the reciprocal of total mass of non-food species (Figure S7 middle).

*
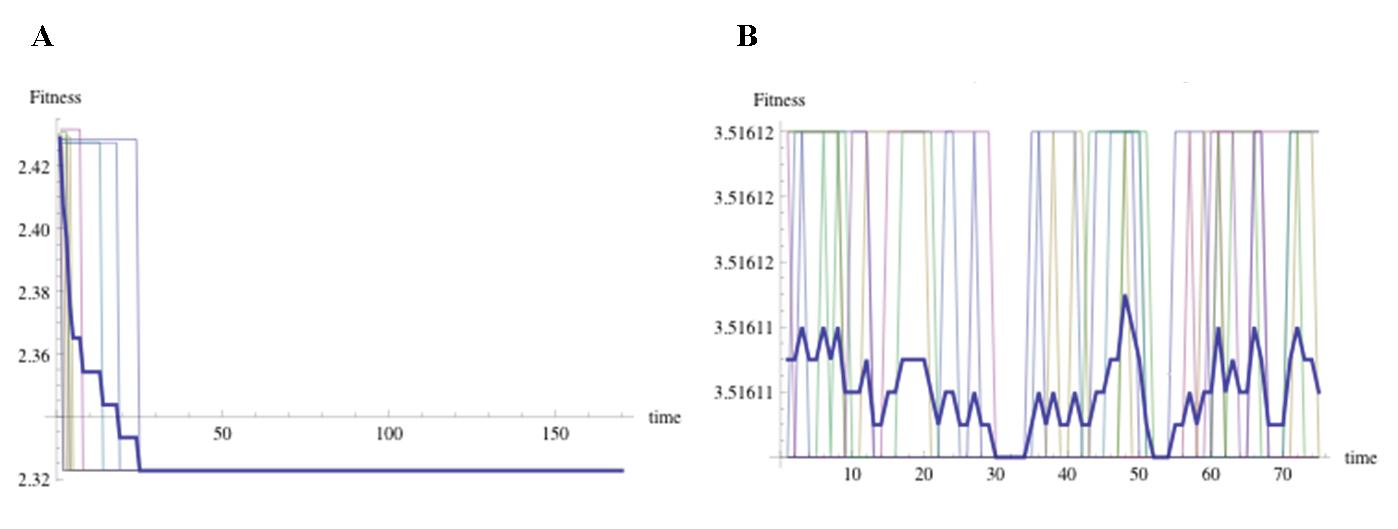
*

Figure S6. Artificial selection for increased non-food mass in networks with inhibition. Narrow lines: fitness (total non-food set mass) of each compartment; thick line: average fitness. The population typically settled down into one equilibrium (A) or fluctuated stochastically or periodically between attractors (B). In both cases selection had little effect. *K* = 0.008, *P* = 0.1, *P’’* = 0.0032, *M* = 4, *B* = 2.


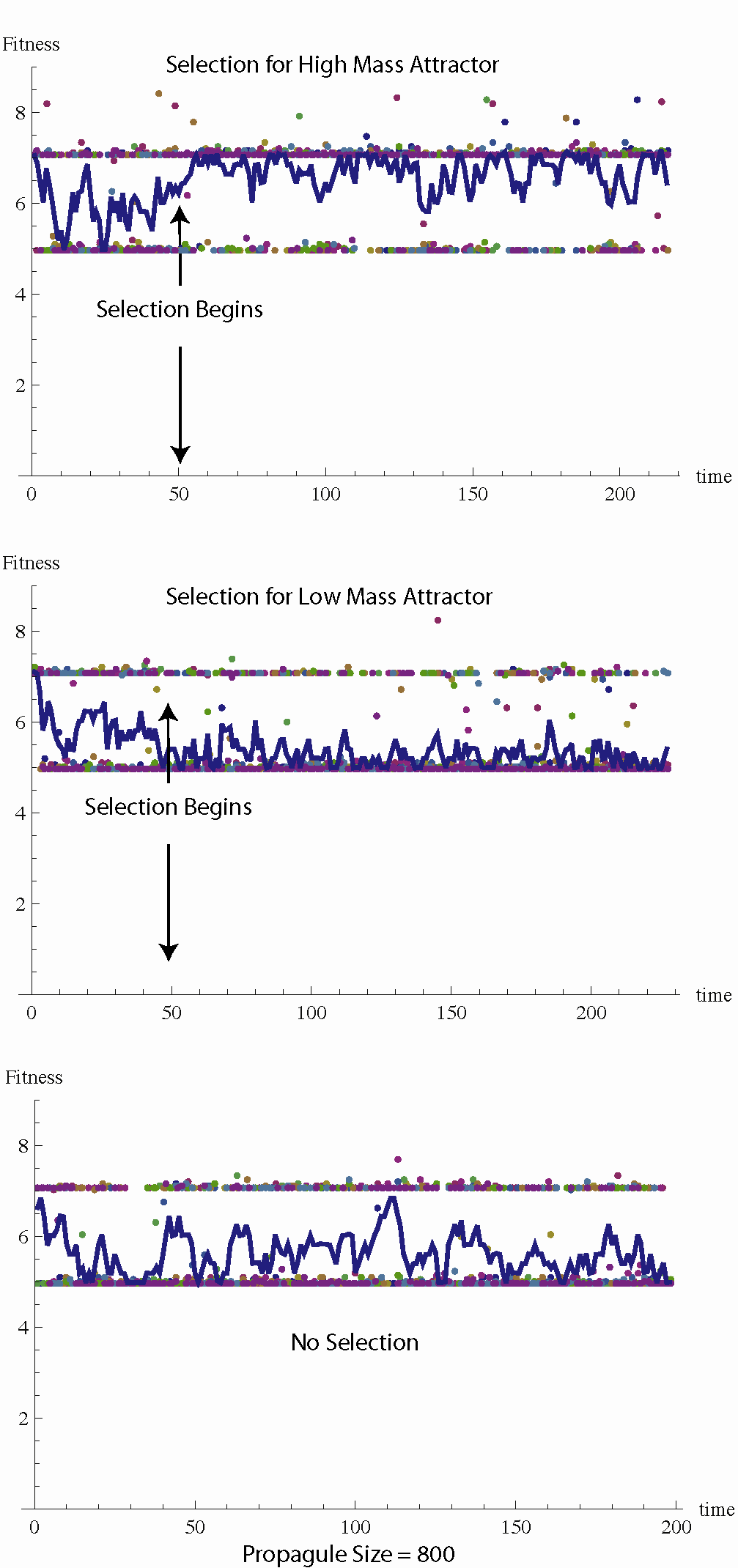


Figure S7. Artificial selection for increased and decreased non-food mass in networks containing a non-food generated viable loop. In both cases there was a response to selection and the population moved towards the high mass or the low mass attractor. Selection started after 50 generations. Dots: fitness (total non-food set mass) of each compartment; thick line: average fitness. (Top) Selection for high mass attractor. (Middle) Selection for low mass attractor. (Bottom) No selection throughout the entire run.

**References**

1. Krishna S (2003) Formation and destruction of autocatalytic sets in an evolving network model. Center for Theoretical Studies, Bangalore, Indian Institute of Science. PhD.

2. Kauffman SA (1986) Autocatalytic sets of proteins. J Theor Biol 119: 1–24.

3. Bagley RJ, Farmer JD, Fontana W (1992) Evolution of a metabolism. . In: Langton CG, Farmer JD, Rasmussen S, Taylor C, editors. Artificial Life II: A Proceedings Volume in the Santa Fe Institute Studies in the Science of Complexity Vol 10. Reading, Massachusetts: Addison-Wesley. pp. 141-158.

4. Hordijk W, Steel M (2004) Detecting autocatalytic, self-sustaining sets in chemical reaction systems. J Theor Biol 227: 451–461.

5. Jain S, Krishna S (1998) Autocatalytic Sets and the growth of complexity in an evolutionary model. Phys Rev Lett 81: 5684-5687.

6. Lifson S (1997) On the crucial stages in the origin of animate matter. J Mol Evol 44: 1-8.

7. Farmer JD, Kauffman SA, Packard NH (1986) Autocatalytic replication of polymers. Phisica D 22: 50-67.

8. Siek JG, Lee L-Q, Lumsdaine A (2002) The Boost Graph Library: User Guide and Reference Manual. New York: Addison-Wesley Professional. 321 p.

9. Bäck T (1996) Evolutionary Algorithms in Theory and Practice Oxford: Oxford Univ Press. 314 p.

1. In an associated paper [3] it is stated that an autocatalytic set is a “set of polymers such that each polymer is produced by at least one catalytic reaction involving only other members of the autocatalytic set”. However, in their Figure 2. a species that catalyses its own formation is considered to be a type of autocatalytic set. Therefore, it appears that they use the same definition as Kauffman [2]. [↑](#footnote-ref-2)
2. The proof itself contains a mistake. Kauffman argues that because any specific longest polymer of length *M* can be constructed in *M*-1 possible end condensation reactions (e.g.: aabb can be constructed in three condensation reactions: aab+a, aa+ba, a+aba), and all reactions can be catalyzed by any of the molecules, the probability that none of the reactions is catalyzed by any of the molecules equals . This implies that as *M* increases, decreases, meaning that the longer the polymer we consider, it becomes less and less likely that its production is not catalyzed. However, this is only true if all end condensation reactions (*M*-1) are possible as all precursors are also catalyzed and if all possible catalysts (*N*) are present, i.e. the probability calculation for one specific longest polymer assumes that all other species are present. This is neither the case nor, as a matter of fact, the goal. [↑](#footnote-ref-3)
3. A note on Lifson’s [6] criticism: in Kauffman’s model, *P* remains constant and so the average number of reactions catalyzed by one molecular species increases rapidly with network size. In Lifson’s [6] interpretation, *P* is inversely proportional to the total number of possible reactions and the average number of reactions that a molecule catalyses is constant. Hordijk and Steel [4] have shown that a linear growth of *P* with system size is sufficient for *RAF* sets to appear with high probability. [↑](#footnote-ref-4)
4. A species is a candidate until it has achieved a concentration above the threshold. Therefore when all species are created in new reactions (except for spontaneous new reactions) they are candidates. [↑](#footnote-ref-5)
